# An open problem: Why are motif-avoidant attractors so rare in asynchronous Boolean networks?

**DOI:** 10.1007/s00285-025-02235-8

**Published:** 2025-06-12

**Authors:** Samuel Pastva, Kyu Hyong Park, Ondřej Huvar, Jordan C. Rozum, Réka Albert

**Affiliations:** 1 Faculty of Informatics, Masaryk University, Botanicka 68a, 60200 Brno, Czech Republic; 2 Institute of Science and Technology Austria, Am Campus 1, 3400 Klosterneuburg, Austria; 3 Department of Physics, Davey Laboratory, Pennsylvania State University, 16802 University Park, Pennsylvania USA; 4 Department of Systems Science and Industrial Engineering, Binghamton University (State University of New York), Engineering Building, 13850 Vestal, New York USA

**Keywords:** Boolean networks, Boolean models, Discrete dynamics, Complex systems, Biomolecular networks, Trap spaces, Stable motif, 94C11, 92C42

## Abstract

Asynchronous Boolean networks are a type of discrete dynamical system in which each variable can take one of two states, and a single variable state is updated in each time step according to pre-selected rules. Boolean networks are popular in systems biology due to their ability to model long-term biological phenotypes within a qualitative, predictive framework. Boolean networks model phenotypes as attractors, which are closely linked to minimal trap spaces (inescapable hypercubes in the system’s state space). In biological applications, attractors and minimal trap spaces are typically in one-to-one correspondence. However, this correspondence is not guaranteed: motif-avoidant attractors (MAAs) that lie outside minimal trap spaces are possible. MAAs are rare and poorly understood, despite recent efforts. In this contribution to the BMB & JMB Special Collection “Problems, Progress and Perspectives in Mathematical and Computational Biology”, we summarize the current state of knowledge regarding MAAs and present several novel observations regarding their response to node deletion reductions and linear extensions of edges. We conduct large-scale computational studies on an ensemble of 14 000 models derived from published Boolean models of biological systems, and more than 100 million Random Boolean Networks. Our findings quantify the rarity of MAAs; in particular, we only observed MAAs in biological models after applying standard simplification methods, highlighting the role of network reduction in introducing MAAs into the dynamics. We also show that MAAs are fragile to linear extensions: in sparse networks, even a single linear node can disrupt virtually all MAAs. Motivated by this observation, we improve the upper bound on the number of delays needed to disrupt a motif-avoidant attractor.

**Supplementary Information:**

The online version contains supplementary material available at 10.1007/s00285-025-02235-8.

## Introduction

Boolean networks are discrete dynamical systems that have been widely and successfully applied to model complex processes, especially in systems biology (Schwab et al. 2020; Rozum et al. 2024; Abou-Jaoudé et al. 2016). They describe the causal logic of regulatory dynamics using *nodes* to represent biomolecular entities (such as genes, proteins, or metabolites) that are connected by *directed edges* representing regulatory influences. Each node (also referred to as variable) can take on one of two states at any given time step—ON (1) or OFF (0)—that is updated according to an *update function* of the node’s incoming regulators’ states.

The nodes and edges that underpin the dynamics of a Boolean network form its *interaction graph*. Signs are often assigned to the individual edges, indicating an activating (+1), inhibiting (-1), or ambiguous (0) influence.^1^ The interaction graph is the primary and best-constrained parameter of the Boolean network: numerous properties can be inferred solely from its structure (Paulevé and Richard 2012). However, many Boolean systems can share the same interaction graph, thus many problems cannot be conclusively solved based on the interaction graph alone.

The dynamics of a Boolean network are affected by how nodes (variables) are selected for update in each time step (Park et al. 2023). Various choices are possible, including updating all variables (*synchronous update*), randomly selecting a single variable to update (*asynchronous update*), or other more complicated schemes involving hidden states, such as the *most-permissive* scheme of (Paulevé et al. 2020). In this work, we focus primarily (but not exclusively) on the asynchronous update scheme.

Typically, the long-term dynamics of a Boolean network (i.e., its *attractors*) are intended to correspond to the biological phenotypes of the system under study. In the context of Boolean networks, attractors are defined as minimal trap sets i.e., the smallest sets of states that are invariant under the network’s dynamics (Schwab et al. 2020; Rozum et al. 2024). There are various efficient methods to identify attractors, despite the NP-hardness of the problem (Mori and Akutsu 2022; Rozum et al. 2021; Beneš et al. 2021; Van Giang and Hiraishi 2021; Trinh et al. 2024).

Attractors alone may be insufficient to answer many practical questions about Boolean networks. For example, one may wish to understand which interventions can or cannot drive the system toward a target behavior (Paul et al. 2019; Cifuentes-Fontanals et al. 2022; Brim et al. 2023), which components of the system contribute to certain phenotypes (Beneš et al. 2022), or which initial conditions lead to which outcomes (Albert et al. 2008). One fruitful approach toward answering these questions is to study how node states reinforce each other to lock a subnetwork of the system into a particular configuration (called a *stable motif*, see Zañudo and Albert 2013). Once reached, these stable configurations confine the dynamics to a subspace in which the constrained nodes are fixed, i.e., to a *trap space* (Klarner et al. 2015). Stable motifs determine the system’s commitment to one phenotype versus another. When a system has multiple stable motifs, the trap spaces nest within one another.

The most deeply nested *minimal trap spaces* must each contain one or more attractors. However, there can be additional attractors that do not lie within any minimal trap space. In this case, the set of minimal trap spaces is called *incomplete* (Klarner and Siebert 2015), and an attractor outside all minimal trap spaces is called *motif-avoidant* (Rozum et al. 2021), as states belonging to the attractor avoid the self-reinforcing network configurations (stable motifs) that result in constraining the dynamics to a minimal trap space. Despite recent efforts (Richard and Tonello 2023; Naldi et al. 2023), motif-avoidant attractors (MAAs) are still poorly understood, especially under asynchronous update. Empirical evidence suggests that motif-avoidant attractors are exceedingly rare in real-world biological networks (Tonello and Paulevé 2023; Trinh et al. 2024), but to the best of our knowledge, this observation has not been quantified before. Despite their apparent rarity, MAAs introduce complications into otherwise straightforward and efficient algorithms for attractor enumeration (Klarner and Siebert 2015; Rozum et al. 2021; Tonello and Paulevé 2023). Therefore, a better understanding of MAAs may lead to significant improvements in Boolean network algorithms.

In this work, we seek to lay out the current state of knowledge about MAAs and discuss some key open problems concerning their presence or absence. Inspired by Tonello and Paulevé (2023) and Naldi et al. (2023), we explore how motif avoidance relates to *node deletion reduction* and to adding a delay to a regulation (*linear extension*). To gain further insight into these phenomena, we conducted multiple large-scale computational studies involving approximately 14 000 networks derived from published biological models and one hundred million Random Boolean Networks (RBNs) (Kauffman 1969). We use these insights to guide the development of some analytical results regarding MAA fragility.

In Sects. 2 and 3, we introduce the necessary formal concepts and recall published results about motif-avoidant attractors. Sect. 4 presents examples of MAAs that challenge our expectations of when motif avoidance should or should not be possible. Sect. 5 explores the rarity of motif avoidance: We find no asynchronous MAAs in published Boolean models of biological systems, even when accounting for a wide array of model inputs and contexts. In RBNs, we show that the likelihood of encountering motif avoidance is non-trivial for dense networks (near $N=K$), but decreases rapidly for sparse networks. In Sect. 6, we employ node deletion reduction and find several MAAs in the reduced versions of two published Boolean models. To the best of our knowledge, these are the first known MAAs in biologically interpretable asynchronous Boolean networks. We also demonstrate that the likelihood of encountering an MAA in a maximally reduced RBN does not depend on the size of the original (non-reduced) network. Finally, Sect. 7 demonstrates that most MAAs can be eliminated by adding a delay node to a single edge, despite the known requirement for $\mathcal {O}(|E|)$ delays in the worst case (Naldi et al. 2023). We derive an improved upper bound for the number of delays needed to eliminate an MAA, present a family of networks that demonstrate that this bound cannot be lowered below |*E*|/4, and identify a simple criterion for when a single delay can destroy an MAA.

## Preliminaries

Boolean networks were introduced by Kauffman (1969) and Thomas (1973) as prototypical models for gene regulatory networks that underlie cell fate decisions (such as those that happen during cell differentiation). In the six decades since their introduction, Boolean models have proven effective in a variety of biological case studies, and the study of ensembles of generic Boolean networks (Random Boolean Networks) (Kauffman 1969) yielded various hypotheses about cell dynamics and evolution.

***Boolean function notation***

Any Boolean function can be expressed algebraically using the logical operators “not” (which we denote by “!”), “or” (which we denote “|”), and “and” (which we denote “ &”). We denote variables with capital letters (e.g. A, B, C, ...) and separate the variable and its function with “,”. We use this notation due to its compactness and because it is frequently used in Boolean network analysis software. Example 2 demonstrates this notation on a simple two-variable Boolean model.

***State transition graph***

Each Boolean system with *N* variables induces a state transition graph (STG) with $2^N$ nodes that represent all possible system states. A directed edge from a node (system state) $S_1$ to another state $S_2$ exists when $S_1$ can be updated in one time step to obtain $S_2$. Under synchronous update, the successor $S_2$ is obtained by applying *all* update functions to $S_1$ in one step, hence each state has a single outgoing transition. Under asynchronous update, each state has between 1 and *N* outgoing edges, with each edge corresponding to the update of exactly one network variable (selected at random). Unless specifically noted otherwise, we assume the asynchronous update scheme throughout this work.

***Trap spaces and succession diagrams***

**Fig. 1:**
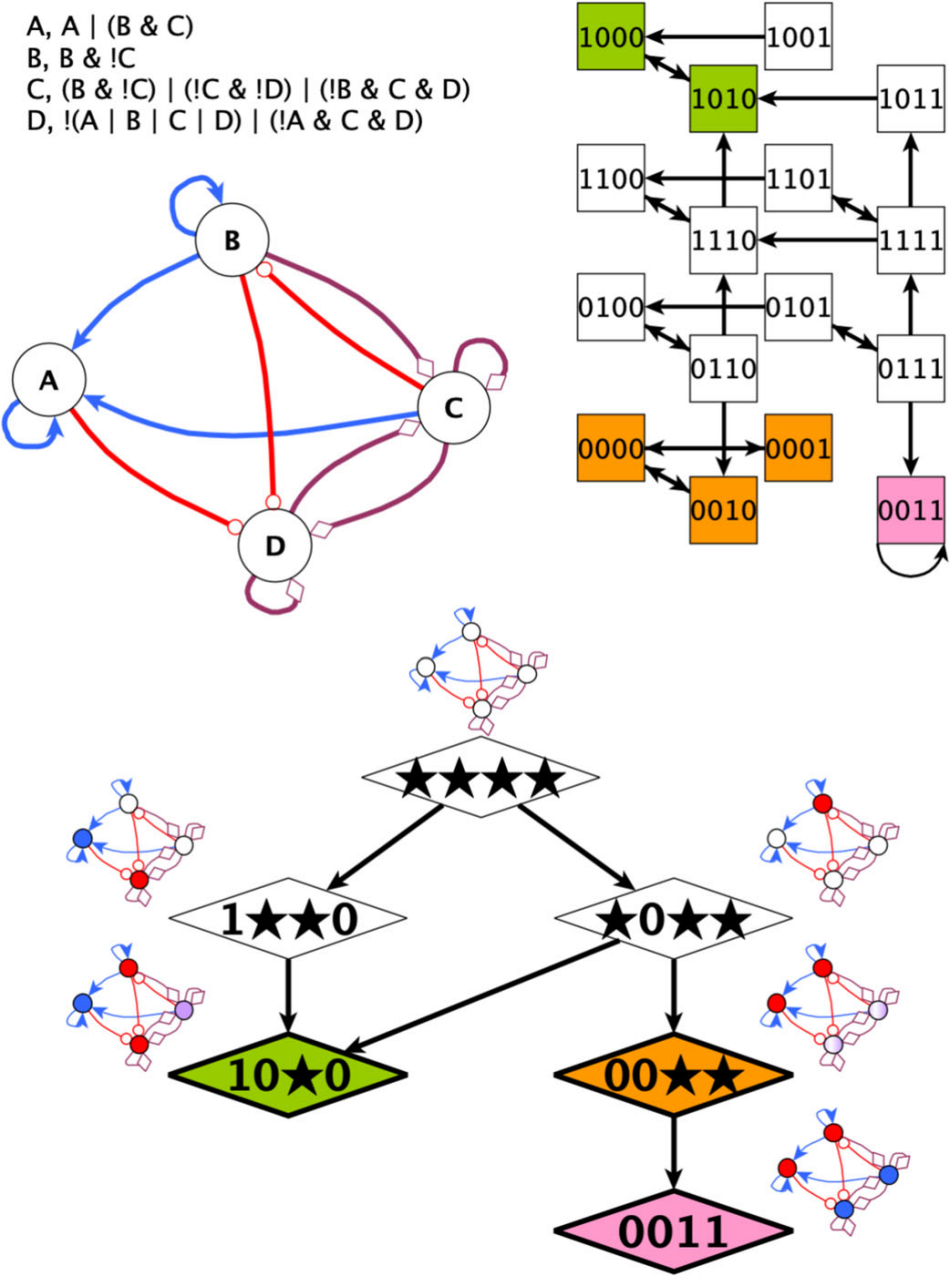
An example Boolean network that has a motif-avoidant attractor. The Boolean network’s interaction graph and update functions are shown in the top left, with blue triangle-tipped arrows representing activation, red circle-tipped arrows representing inhibition, and purple diamond-tipped edges representing dual influences (either activation or inhibition, depending on context). The state transition graph of the system is shown in the top right, with attractors highlighted by color. For clarity, we only show self-edges on fixed points. On the bottom, the succession diagram is shown. Here, each node in this acyclic graph represents a trap space in the state transition graph, and is depicted alongside a cartoon of the system state it represents, with constrained variables colored blue (for ON) or red (for OFF), and variables that oscillate in purple (the intensity of the color distinguishes two different attractors). Edges denote set inclusion. The smallest trap space containing each attractor is colored accordingly; if that trap space is not a leaf node, i.e., a minimal trap space (as in the case of the orange attractor), the attractor is motif avoidant

A Boolean network *subspace* is a set of states that is characterized by a subset of network variables fixed to constant values. In other words, a subspace is a hypercube in the state space of a Boolean network. Subspaces are typically denoted by *N* symbols “0” (fixed to OFF), “1” (fixed to ON), or “*” (unconstrained), each giving the requirement for one variable. For example, the subspace denoted $$0*1*$$ corresponds to the set of states $$\{ 0010,0011,0110,0111 \}$$. A subspace is called a *trap space* if its states form a trap set: a set that cannot be escaped by the network dynamics. Inclusion *minimal trap spaces* are of particular interest here, as they represent parts of the state space that cannot be escaped, but that also cannot be further constrained into a smaller trap space. Each minimal trap space is determined by a cyclic influence network among the variables that are fixed in the trap space; the minimal strongly connected components of this network are called stable motifs. Stable motifs can be identified, for example, using a hypergraph representation of the update functions (Albert and Othmer 2003; Zañudo and Albert 2013; Rozum et al. 2021). Figure 1 illustrates multiple types of attractors and trap spaces. The minimal trap space $$10**$$ (shown in green) is due to the combination of two stable motifs, namely the self-sustained ON state of $$\texttt {A}$$ and the self-sustained OFF state of $$\texttt {B}$$. Within the trap space $$00**$$, the regulatory cycle between $$\texttt {C}$$ and $$\texttt {D}$$ forms a stable motif that sustains the ON state of both variables, leading to the minimal trap space 0011. Importantly, trap spaces do not change with the chosen update scheme, in contrast to the state transition graph.

Trap spaces can nest within one another, leading to a complex branching structure, called a *succession diagram* (Rozum et al. 2021) (see Fig. 1), that encodes which stable motifs cause the system to commit to one fate or another. The succession diagram is a directed acyclic graph whose nodes are trap spaces (with fixed values percolated through the logical update functions) and whose edges represent inclusion. Thus, the leaf nodes of this graph are in one-to-one correspondence with the minimal trap spaces.

***Motif-avoidant attractors***

The *attractors* of a Boolean system are the minimal trap sets of the dynamics. In other words, they are the terminal strongly connected components of the STG. They are divided into two types: *point attractors* (also called fixed points or steady states), which contain only one system state, and *complex attractors*, which contain more than one state (in synchronous update, these are always cycles; in asynchronous update, these can also be more complex components). A *motif-avoidant attractor* (MAA) is a complex attractor that does not lie within a minimal trap space. By this definition, the smallest subspace containing a motif-avoidant attractor is not a minimal trap space. Thus, either there exists a state in the subspace that has a transition out of the subspace, or the subspace contains a minimal trap space that is disjoint from the MAA. An example of the second case is given in Fig. 1. Detecting MAAs is computationally difficult, and requires identifying both the attractors (Mori and Akutsu 2022) and all minimal trap spaces (Moon et al. 2022).

***Update schemes and their effect on attractors***

The states of variables in a Boolean network can be updated in each time step according to various schemes. The earliest, and among the most popular, is the synchronous update scheme, in which all variables are updated at every time step (Kauffman 1969). While this scheme has computational and analytical advantages, it can also give rise to oscillations that depend on perfect synchronization of variables (Park et al. 2023). This can be undesirable when these variables model inherently stochastic processes, such as gene transcription. The most popular alternative, and our focus in this work, is the general (also called stochastic) asynchronous update scheme, in which a single, randomly chosen variable is updated at each time step. In the even more general random set update scheme, a randomly selected subset of the variables is updated at each time step. The most permissive update scheme possible is the aptly named Most Permissive Boolean Network (MPBN) framework (Paulevé et al. 2020), which introduces intermediate “increasing” and “decreasing” hidden states to allow a wider variety of transient behavior than is possible with other update schemes. All possible synchronous and asynchronous state transitions (and more) are permitted within the MPBN scheme.

While the trap spaces (including fixed points) of a Boolean network are independent of the selected update scheme, the number and characteristics of oscillatory attractors do depend on how variables are updated. Such attractors observed under one type of update may disappear when using a different update scheme. In particular, a Boolean network may have MAAs under one update scheme but not another.

In prior work, we have analyzed the impacts of update scheme in Boolean models from the literature (Park et al. 2023) and in random models (Rozum et al. 2021). We and others have observed that motif-avoidant attractors are fairly common in synchronously updated Random Boolean Networks (Klemm and Bornholdt 2005), and are a significant contributor to the substantially higher number of attractors observed under synchronous update (Kaufman et al. 2005) than under asynchronous update (Greil and Drossel 2005; Rozum et al. 2021). They have also been observed in several models of cell processes (Park et al. 2023). For example, a model of the cell cycle of neuroblastoma cancer cells (Dahlhaus et al. 2016) and a model of the interactions of 9 cell cycle transcription factors (Orlando et al. 2008) each exhibit a biologically substantiated motif-avoidant attractor under synchronous update; this attractor disappears under asynchronous update. This fragility to asynchronicity is typical of synchronous motif-avoidant attractors: the simple feedback loop system whose update functions are given in Example 1 illustrates this.

### [Style3 Style3]Example 1

(A synchronous motif-avoidant attractor destroyed by asynchronous update) Consider the Boolean update functions below, in which A updates to take on the state of B (written A, B) and vice versa. The synchronous attractor $01\rightarrow 10\rightarrow 01$ is motif-avoidant (it avoids the trap spaces 00 and 11). This attractor vanishes under asynchronous update because changing the state of either variable in 01 or 10 leads to one of the two point attractors.
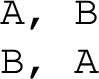


Generally, the more transitions that are possible in a given update scheme, the less likely MAAs are to occur: in the extreme case, the MPBN framework ensures that MAAs do not occur at all. With respect to MAA occurrence, the asynchronous update scheme represents a widely used but poorly understood middle ground between synchronous update and the MPBN framework.

***Node deletion reduction***

A node of a Boolean network that does not self-regulate can be deleted by substituting its update function into the functions of the nodes regulated by it. The reduced Boolean system obtained after this deletion has been proven (Naldi et al. 2011; Veliz-Cuba 2011; Tonello and Paulevé 2023) to preserve certain features of the original system’s attractor repertoire. In particular, all point attractors are preserved, and complex attractors of the original system map to one or more complex attractors of the reduced system. Naldi et al. (2011) proved that any transition in the reduced system corresponds to at least one transition in the original system, and that the transitions starting from representative states (in which the update of the to-be-reduced node does not change its state) are preserved in the reduced system. Node deletion can be repeated while self-regulation-free variables exist in the system; the preservation of the point attractors and possible emergence of new complex attractors is maintained regardless of the number of deleted variables. It is possible that a complex attractor that emerges due to node deletion is a motif-avoidant attractor.

***Linear extensions***

Linear extension of an edge $\texttt {X} \rightarrow \texttt {Y}$ means the edge is replaced with an intermediary node (network variable) $\texttt {Z}$ and two edges $\texttt {X} \rightarrow \texttt {Z} \rightarrow \texttt {Y}$. The update function of $\texttt {Z}$ is simply $\texttt {X}$ (i.e. $\texttt {Z}$ propagates the value of $\texttt {X}$), and $\texttt {X}$ is replaced by $\texttt {Z}$ in the update function of $\texttt {Y}$. If such node $\texttt {Z}$ already exists in a Boolean network, we call it a *linear node* or a linear component (Naldi et al. 2023). The deletion of a linear node is a special case of network reduction, thus the previously identified properties of network reduction apply to it. In particular, deleting a linear node may lead to the emergence of a motif-avoidant attractor. Furthermore, the addition of a linear node can eliminate a motif-avoidant attractor, and under certain well-defined conditions (as we discuss in more detail in Sect. 3), this guarantees the network has no MAAs.

***Biological Boolean network ensemble***

To empirically evaluate the properties of motif-avoidant attractors in real-world models, we consider the *Biodivine Boolean Models* (BBM) dataset (Pastva et al. 2023), which is to the best of our knowledge the largest collection of biologically relevant Boolean networks. The dataset covers 230 models aggregated from various sources, including The Cell Collective (Helikar et al. 2012), GINsim (Gonzalez et al. 2006), Biomodels (Malik-Sheriff et al. 2020), the COVID-19 Disease Map project (Ostaszewski et al. 2020), other literature reviews (Kadelka et al. 2024), and manually curated papers. Most of these models describe within-cell processes and cells’ interaction with their environment or with pathogens. For each model, we infer the interaction graph from the semantic properties of the model’s update functions, as some networks are originally published with redundant interactions or incomplete sign annotations. Most of these models contain input nodes, which denote information from the environment or factors outside of the scope of the model. As the values of input nodes significantly influence the attractor repertoire of Boolean models (Park et al. 2023), we consider all input valuations when possible, which yields over $2^{100}$ model variants. In settings where this is impossible, we consider up to 128 unique, but randomly chosen valuations for these constant inputs, which yields 14 136 distinct model variants.

***Random Boolean network ensembles***

We also consider a collection of N-K Random Boolean Networks (RBNs) (Kauffman 1969), where each of the *N* nodes receives *K* edges from randomly selected nodes. The (quenched) update functions are chosen such that each of the $2^K$ input combinations yields an output of 1 with probability *p*. Tuning *p* can produce an order-to-chaos transition (Aldana et al. 2003) at $2Kp(1-p)=1$ (in the thermodynamic limit, when $N\rightarrow \infty$). Ensembles where *p* satisfies this equation are called *critical*. In this work, we study several large critical ensembles of RBNs across a wide range of *N* and *K*. Specifically, we consider ensembles of small RBNs with $N \in [4, \ldots , 10]$ together with the full range of admissible *K*, and large RBNs with $N \in [20,30,40]$ and $K \in [2, \ldots , 5]$.

To achieve reasonable statistical significance for each ensemble (i.e., combination of *N* and *K*), we sample random networks using pystablemotifs (Rozum et al. 2022) until we discover 1000 networks with motif-avoidant attractors.^2^ Here, we are not concerned with the number of such attractors in individual networks, only whether *at least one* motif-avoidant attractor exists. Overall, we tested more than 100 million random networks. The number of sampled networks across the ensembles is indicated in Table B1 in the Appendix.

***Sensitivity and effective connectivity of Boolean functions***

Canalization refers to the ability of a biological system to buffer fluctuations in genetic and environmental signals. It is reflected in the structure of Boolean network models of biological processes, and is an important property for understanding emergent robustness. There are many measures of canalization in Boolean networks; here, we focus on two: sensitivity and effective connectivity.

The sensitivity of a Boolean function is defined as the average change in the function’s output in case of a change in the value of one of its inputs (Shmulevich and Kauffman 2004). A Boolean network can be characterized by the average sensitivity of its functions. The expected average sensitivity of critical RBNs equals 1 (Shmulevich and Kauffman 2004). Multiple studies indicated that the average sensitivity of ensembles of biological Boolean networks is very close to 1 (Subbaroyan et al. 2022; Kadelka et al. 2024).

The effective connectivity of a Boolean function is defined as the mean number of input values required to determine the output for each input state (Correia et al. 2018). An effective connectivity that is significantly smaller than the average in-degree indicates that the system is canalizing, i.e., certain variables play a disproportionate role in determining a stable dynamic outcome (Manicka et al. 2022; Costa et al. 2023). Studies of published Boolean models indicate that their effective connectivity is smaller and less heterogeneous than the average in-degree in the models’ interaction networks (Manicka et al. 2022; Costa et al. 2023).

***Tools and algorithms***

We provide a reproducibility artefact at https://doi.org/10.5281/zenodo.13860057 which contains all code and data that is necessary to replicate the results of this paper. As stated previously, the artefact uses pystablemotifs (Rozum et al. 2022) to generate the ensembles of critical RBNs. For structural manipulation of networks (reductions, linear extensions, etc.), we rely on AEON.py (Beneš et al. 2020, 2022). Finally, to detect motif-avoidant attractors and their associated trap spaces, we use the symbolic techniques implemented in AEON.py in combination with the succession diagrams generated by biobalm (Trinh et al. 2024). AEON.py uses binary decision diagrams (BDDs) to symbolically encode the STG of a Boolean network, such that even large state spaces (of more than 30 variables) can be explored efficiently. Furthermore, BDDs can be used to analyze and transform the Boolean network’s update functions, for example, to obtain the underlying interaction graph or to simplify a Boolean network after a reduction. Meanwhile, biobalm uses answer set programming to generate the network’s succession diagram, which can be used to identify motif avoidance and enclosing trap spaces of motif-avoidant attractors, but also to speed up attractor computation in general.

## Theoretical results relevant to motif-avoidant attractors

As proven by Richard (2010), under asynchronous update, the existence of a non-positive cycle (determined by the product of the +1, -1, 0 edge signs) in the interaction graph is a requirement for a complex attractor (sustained oscillation). In addition, in order to have a minimal trap space that is not trivially the entire state space, a Boolean network must have at least one non-negative cycle in its interaction graph (Richard and Comet 2007). By similar logic, a network needs to have multiple attractors in order to have a motif-avoidant attractor, which also requires the presence of a non-negative cycle. Thus, Boolean networks with no cycles, only positive cycles, or only negative cycles cannot have motif-avoidant attractors (MAAs).

### Separating and trap-separating networks

Richard and Tonello (2023) derive necessary conditions for the interaction graph of a Boolean network such that the network is not *separating* (i.e., there is a pair of attractors whose smallest subspaces overlap) or not *trap-separating* (i.e., there is a pair of attractors whose smallest trap spaces overlap). A motif-avoidant attractor and the attractor determined by the avoided motif are not trap-separating, and may not be separating (i.e., they could be in the same subspace, as we will see in examples in later sections). Richard and Tonello prove that if the Boolean network is not trap-separating, then its interaction graph has a path from a non-positive cycle to a non-negative cycle. They also prove that if the Boolean network is non-separating, three statements are true about the interaction graph: (i) it has either a non-negative cycle that intersects a non-positive cycle or a cycle in which at least one edge is ambiguous, (ii) it has at least two non-positive cycles or an ambiguous cycle with at least two ambiguous edges, and (iii) at least two vertices must be removed to destroy all the non-negative cycles.

### Boolean networks with L-cuttable interaction graphs

The interaction graph of a Boolean network admits a linear cut when a linear node (a node with in-degree and out-degree one) occurs in each cycle (including self-edges) and in each path from a node with multiple targets to a node with multiple regulators. Naldi et al. (2023) proved that in a Boolean network whose interaction graph admits a linear cut the attractors are in a one-to-one relationship with minimal trap spaces. As a consequence, these L-cuttable networks do not have motif-avoidant attractors. In addition, in an L-cuttable network, all states reachable from a canonical state (a system state in which each linear node has the same state as its sole regulator) by updating any set of nodes are also reachable by updating one node at a time. Naldi et al. propose that the unreachability of certain states under asynchronous update is due to unsatisfiable circular requirements on the order of update of the nodes. An example of such an unsatisfiable circular requirement is “In order to reach system state *Y* from system state *X*, $\texttt {A}$ needs to be updated before $\texttt {B}$ (because the updated state of $\texttt {A}$ is needed to allow the state change of $\texttt {B}$), $\texttt {B}$ needs to be updated before $\texttt {C}$, and $\texttt {C}$ needs to be updated before $\texttt {A}$ (because the un-updated state of $\texttt {A}$ is needed to allow the state change of $\texttt {C}$)”. Naldi et al. observe that such unsatisfiable constraints arise from feedback loops or incoherent feed-forward loops in the interaction graph. These conflicts can be resolved by adding linear extensions to edges until the interaction graph admits a linear cut. In the worst case, all the edges need linear extensions.

### Linear extensions

Each linear node added in a linear extension of an edge can be thought of as a delay node, an intermediary between its parent node (the starting node of the original edge) and the end node of the original edge. Each delay node serves as a memory of the un-updated state of its parent node. If each edge has a delay node, both states of each variable are represented in the system, thus unsatisfiable requirements on the update order are no longer possible.

A key feature of a motif-avoidant attractor is the unreachability of certain state(s) of the enclosing subspace from the states visited by the attractor. While a maximal extension is sufficient to eliminate any motif-avoidant attractor by ensuring the reachability of all states in the subspace, the gain in reachability needed to eliminate a specific motif-avoidant attractor may be achieved with fewer extensions. Consider a system with a 2-variable MAA combined with a minimal trap space 11.

#### [Style3 Style3]Example 2

(Prototypical 2-variable motif-avoidant attractor) The update functions of the sole network with two variables that exhibits a motif-avoidant attractor.
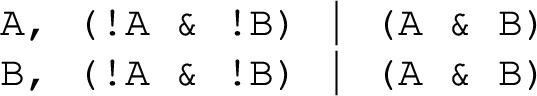


In Example 2 the reachability of the state 11 from the state 00 has an unsatisfiable requirement: $\texttt {A}$ needs to be updated before $\texttt {B}$ to allow its state change, and $\texttt {B}$ needs to be updated before $\texttt {A}$ to allow its own state change. A full linear extension of this system requires 4 delay nodes. Yet, the unsatisfiable requirement can be eliminated by a single delay node. For instance, when adding a delay node $\texttt {C}$ to the $\texttt {A} \rightarrow \texttt {B}$ edge, the requirement for the reachability of 111 from 000 becomes “$\texttt {A}$ needs to be updated before $\texttt {B}$, and $\texttt {B}$ needs to be updated before $\texttt {C}$”, which is now satisfiable due to the newly added delay node.

The literature discussed in this section has produced various sufficient conditions for the elimination of motif avoidance; these conditions are often quite strict, and are not met for many networks that do not contain MAAs. Motivated by this observation, we sought to investigate the related questions of what makes motif avoidance appear and what is the minimal number of delays that eliminates a motif-avoidant attractor. Our investigation was small-scale at first. We performed an exhaustive search to enumerate the possible MAAs of 3-variable Boolean networks. We dedicated a significant effort to identifying conditions that guarantee or rule out motif avoidance. In the next section, we present a sampling of examples to illustrate the challenges of this investigation.

## Motif-avoidant attractors defy expectations

**Table 1:** Summary of the key computational and analytical results of this work

Boolean network type	Key results	Section
Prototypical examples of motif avoidance	MAAs do not require non-monotonic functions or negative self-edges	§4.1
	Low canalization favors, but is not required for motif avoidance	§4.2
	Six prototypical MAAs are destroyed by a single linear extension, the remaining one is destroyed by a combination of two linear extensions	§7.2
Reduced versions of published Boolean models	We observed 9 MAAs derived from two models	§6.2
	All 9 MAAs are destroyed by a single linear extension	§7.2
Sparse critical RBNs	The observed frequency of MAAs is less than 0.04% when K $=$ 2 and N>10	§5.2
	All MAAs are destroyed by a single linear extension for N=10 and K=2	§7.3
Dense critical RBNs	The observed frequency of MAAs is 1.6% for N $=$ K $=$ 10	§5.2
	More than 80% of MAAs are destroyed by a single linear extension	§7.3
Maximally reduced RBNs	The observed MAA frequency is 0.48% if the original networks have K $=$ 2 and 10 $\le$ N $\le$ 40	§6.3
Any Boolean network in which the smallest trap space that contains an MAA and a minimal trap space has M free variables, the MAA has M oscillating variables, and is at minimal Hamming distance 1 from the minimal trap space	A single linear extension can eliminate the MAA	§7.4
Boolean networks with a star-shaped MAA (Example 10)	The number of delays necessary and sufficient to eliminate the MAA is $\frac{N^2}{4}$ for even *N* and $\frac{N^2 - 1}{4}$ for odd *N*	§7.4

We formulated various conjectures about the requirements for motif avoidance and then found counterexamples for each conjecture. The examples in Subsection 4.1 are representative of natural expectations regarding motif-avoidant attractors that are then contradicted by reality. Subsection 4.2 presents minimal patterns in the state transition graph that correspond to MAAs. In general, the MAA can appear at any level of a succession diagram, in which case these examples would be parts of larger systems.

In addition to the examples and counterexamples discussed in this section, we conducted various large-scale computational studies to better characterize the emergence of MAAs. We summarize our key findings in Table. 1. We discuss these results in detail in this and subsequent sections.

### Counterexamples to conjectures about motif avoidance

The interaction graphs and state transition graphs of the systems presented in this subsection are shown in Figure B2 in the Appendix. To focus on the MAA in these examples, we combine them with a single-state minimal trap space (i.e., a point attractor).

#### [Style3 Style3]Example 3

(Simple functional forms) This example shows that MAAs can exist even in Boolean networks with locally monotonic and nested canalizing update functions.
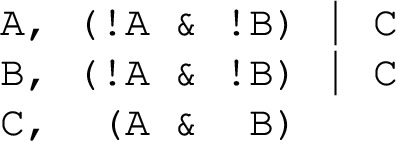


This system has a minimal trap space 111 and a motif-avoidant attractor that visits 3 states of the $$**0$$ subspace. The fourth state of the subspace, 110, has a transition to the minimal trap space 111.

It is well-documented that a regulator’s direct effect on a target tends to be monotonic in biological networks, and that multiple regulators tend to form a hierarchy in which strong (canalyzing) regulators override the weaker regulators’ effect, forming nested canalizing functions (Harris et al. 2002; Jarrah et al. 2007; Subbaroyan et al. 2022; Kadelka et al. 2024). Examples 3 and 4 show that these strong restrictions on the update functions do not rule out motif-avoidant attractors.

#### [Style3 Style3]Example 4

(No need for negative self-edges) Although negative cycles are necessary to have MAAs, negative self-edges are not.
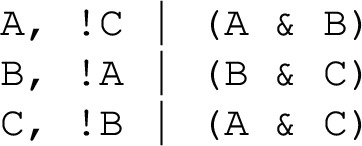


This Boolean network has a minimal trap space 111 and a motif-avoidant attractor that forms a cycle of 6 states. This MAA would be preserved under synchronous update as well as random set update, because the functions do not allow the state change of multiple variables.

Furthermore, it is not true that the minimal trap space is always reachable from the non-attractor states of the MAA subspace. That is, to escape the motif-avoidant attractor, it may be necessary to perturb the network to a state outside the MAA subspace:

#### [Style3 Style3]Example 5

(Temporary escape from the subspace) One way in which a subspace that contains an MAA can be non-trapping is the existence of a trajectory that leaves and then reenters the subspace.
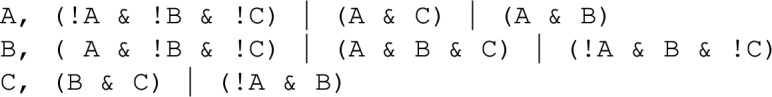


The Boolean network has a minimal trap space 111 and a motif-avoidant attractor that visits the states 000, 100, and 110. The fourth state of the $$**0$$ subspace, 010, is the starting point of the path $$010 \rightarrow 011 \rightarrow 001 \rightarrow 000$$ (see Figure B2 in the Appendix).

Finally, just as multiple asynchronous attractors can appear in one minimal trap space, a single subspace can contain more than one motif-avoidant attractor. That is, the smallest trap space containing an MAA may also contain an additional MAA:

#### [Style3 Style3]Example 6

(Two independent MAAs can coexist in the same enclosing trap space) 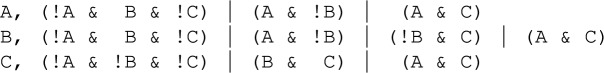


The Boolean network has a minimal trap space 111, a motif-avoidant attractor that visits the states 000, 001, 011, and a second MAA that visits the states 100, 110, 010.

### Prototypical motif avoidance in small networks

The system in Example 2 represents the unique 2-variable MAA (up to relabeling and negation). For a more systematic investigation of MAAs we aimed to identify all types of MAAs in 3-variable systems. We considered a trap space 111 and determined minimal patterns in the state transition graph that can correspond to an attractor that avoids 111. We identified 6 prototypical (template) MAAs, shown in Figure B3 in the Appendix. One of these MAAs was already shown as Example 4, indicating that monotonic and canalizing functions, or the lack of self-inhibition, do not rule out motif avoidance.

Next, we asked whether these prototypical MAA-containing systems have a lower-than-expected degree of canalization. The systems that generate the 2-variable and 3-variable MAAs involve eight unique Boolean functions (up to relabeling and negation). We determined the average sensitivity and effective connectivity of these functions (see Table 2). We found that the sensitivity of the MAA-containing systems is significantly higher than the sensitivity of 1 shared by critical RBNs (Shmulevich and Kauffman 2004) and ensembles of published Boolean models (Subbaroyan et al. 2022; Kadelka et al. 2024). Furthermore, we found that the effective connectivity of the prototypical MAA-containing systems is higher than the effective connectivity of critical 3-variable RBNs, and significantly higher than the observed effective connectivity of 1.202 of published biological Boolean networks (Costa et al. 2023). In summary, in these prototypical MAA-containing systems, more inputs are essential than expected, and there is less canalization than expected.

**Table 2:** Two measures of canalization in the eight unique functions (up to relabeling and negation) we observed in prototypical small networks with MAAs

Rule	Sensitivity	Effective Connectivity
A & B $\mid$ !A & !B	2	2
A $\mid$ B & C	1.25	1.5625
!A & !B $\mid$ A & B & C	1.75	2.125
A & !B $\mid$ !B & !C $\mid$ A & C	1.5	2
A & B $\mid$ B & C $\mid$ A & C	1.5	2
A & !B $\mid$ !A & !C $\mid$ A & B & C	1.75	2.125
!A & !C $\mid$ !B & !C $\mid$ A & B & C	2	2.25
A & B & C $\mid$ !A & !B & !C	1.5	2.25
Avg. of above examples (k=3 only)	$\sim 1.607$	$\sim 2.045$
Avg. k=3 critical ensemble	1	$\sim 1.348$
Avg. previously reported in biological ensembles	1.001 (Kadelka et al. 2024)	1.202 (Costa et al. 2023)

Seeing the diversity of motif-avoidant attractors in small Boolean networks, we followed up with extensive computational studies of Boolean network ensembles, as described in the next sections.

## Motif-avoidant attractors are surprisingly rare

Though motif avoidance in asynchronously updated Boolean networks is known to be more rare than in synchronously updated networks (see Sect. 2), the frequency of asynchronous motif avoidance has not previously been studied systematically. Here, we quantify how common MAAs are under asynchronous update in two widely used network ensembles.

### Motif avoidance in real-world Boolean networks

We performed an extensive computational study of published Boolean models, analyzing all models from the BBM dataset (Pastva et al. 2023). Note that most of these models contain input nodes with constant values. In many cases, only a small subset of the combinations of input values was explored by the model’s authors, and many models have not been checked for motif-avoidant attractors at all. Even though the model behavior has not always been biologically confirmed across all input valuations, here, we still consider all input valuations as relevant, since they represent models that can be linked to real biological processes.

When possible, we use the symbolic “colored” representation in AEON.py (Beneš et al. 2020, 2022) to check all models in the BBM dataset across all input valuations for MAAs. This technique was successful for 221/230 models, accounting for more than $2^{100}$ input valuations overall. Computation for the remaining 9 models exceeded the 24-hour timeout during reachability analysis. These are large models with a high number of input nodes, leading to a wide range of possible minimal trap spaces that could not be compactly encoded in the “colored” representation. For these models, we use randomly sampled unique input valuations (see Sect. 2) and check them for MAAs using biobalm (Trinh et al. 2024), which scales better for large networks when the input values are fixed. Together, this accounted for 1004/1026 input valuations across the remaining 9/230 models.^3^*No motif-avoidant attractor was found in any of these models.* The 95% confidence interval for the proportion of biologically realistic models with MAAs can be calculated via the Wilson interval $\frac{m+2}{n+4}\pm \frac{2}{n+4}\sqrt{1+n\sigma ^2}$, where n=230 is the sample size, m=0 is the number of MAA-containing networks, and $\sigma ^2=0$ is the sample variance, yielding $[0, 1.7\%]$.

We tested whether the lack of MAAs is due to L-cuttability. Only 9/230 models^4^ in the BBM dataset have an L-cuttable interaction graph when considering input nodes as variables, but ignoring their self-regulations, as these alone cannot cause MAAs to appear. To evaluate the simplification due to fixed input values, for each model from the BBM dataset, we tested all input valuations if there are fewer than seven inputs or sampled 128 unique constant input valuations. This process results in 14 136 model variants. We then tested their L-cuttability after the constant values were percolated into their target functions. The interaction graphs are indeed greatly simplified: 5386/14136 of the instances percolate to an empty graph, and 1277 of the remaining 8750 non-trivial graphs are L-cuttable. Taking the effect of constant nodes into account thus greatly increases the number of L-cuttable interaction graphs (to 47%), but not enough to account for the complete lack of MAAs.

### Motif avoidance in random N-K networks

To expand the search, we also considered ensembles of critical Random Boolean Networks according to the N-K model (Kauffman 1969), sampled using pystablemotifs (Rozum et al. 2022). These ensembles are described in more detail in Sect. 2. Ensembles of critical Random Boolean Networks are comparable to ensembles of published biological Boolean models in that the average sensitivity of both ensembles is very close to 1 (Subbaroyan et al. 2022; Kadelka et al. 2024).

**Fig. 2:**
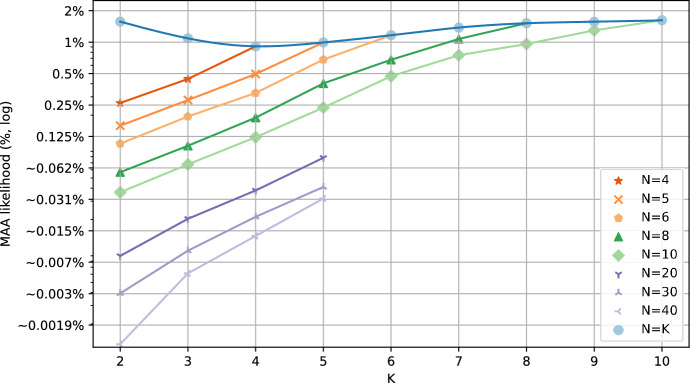
The likelihood of encountering a network with a motif-avoidant attractor within the ensembles of critical N-K random Boolean networks. Note that the likelihood axis is logarithmic. The blue trend-line shows the likelihood for maximally dense networks where $N=K$, the remaining trend-lines show fixed network sizes with varying *K* (for large networks, the data only cover $K \in [2,3,4,5]$). The 95% confidence intervals (Wilson score intervals) for all points are smaller than the displayed points

Figure 2 shows the likelihood of encountering a network with a motif-avoidant attractor in each of the tested ensembles (excluding $N=7$ and $N=9$ for better visual clarity). Notice that even for dense networks of non-trivial size (e.g. $N=K=10$), the MAA likelihood is only $\sim \!1.6\%$. Furthermore, the maximum MAA likelihood for each *N* is achieved when $N=K$. For a fixed value of *K*, the MAA likelihood decreases very quickly with growing *N*. For $K=2$, the MAA likelihood is less than $0.04\%$ when $N \ge 10$ and well below $0.001\%$ for $N \ge 20$. Biological networks are known to be sparse and contain at least tens of nodes (in the BBM dataset, the average *K* is $\sim \!\! 2.3$ and the average *N* is $\sim \!\! 58.01$). Therefore, a contributor to the lack of MAAs in the BBM models may be that in sparse networks with asynchronous update, the possibility of creating an MAA by chance is simply too small.

## Intensifying the search for motif-avoidant attractors by reducing networks

As can be seen in our results presented in Sect. 5.2 and the examples above, all the motif-avoidant attractors we have identified involve a dense network or subnetwork. Such dense subnetworks are very rare in Boolean models. As we did not find any MAAs in the published Boolean models, we propose that reducing networks (by deleting nodes, as described in the Preliminaries) may be one way that MAAs may arise in practice. This proposition is supported by the previous result (Naldi et al. 2023) that the deletion of linear nodes destroys L-cuttability, thus enabling motif avoidance. Yet, we found that node reduction can also eliminate a motif-avoidant attractor by transforming it into a regular complex attractor. We start this section with examples, then we present the results of our search for MAAs in reduced versions of published Boolean models and in maximally reduced Random Boolean Networks.

### Motif avoidance is a non-local property: node reduction can either create or eliminate it

Node deletion reduction preserves point attractors, and complex attractors of the original system map to one or more complex attractors of the reduced system (Naldi et al. 2011; Veliz-Cuba 2011). It is possible that a complex attractor created by node deletion is a motif-avoidant attractor.

#### [Style3 Style3]Example 7

(Reducing a linear node can create an MAA) The MAA created by reducing node $\texttt {C}$ is identical to that of Example 2. It visits three states and avoids the minimal trap space 11.
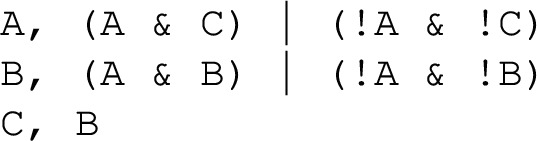


Conversely, we found that node deletion reduction can lead to the emergence of a stable motif, which traps a motif-avoidant attractor into a minimal trap space.

#### [Style3 Style3]Example 8

(Reduction can eliminate motif avoidance) The Boolean network below has a minimal trap space 111 and a motif-avoidant attractor that visits 6 states.
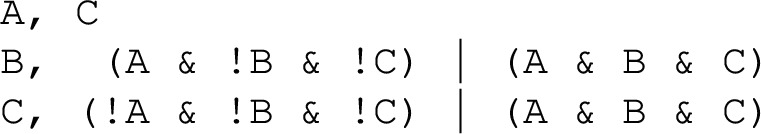


When reducing node $\texttt {A}$, the term (C & !B & !C) in the function of $\texttt {B}$ evaluates to 0.
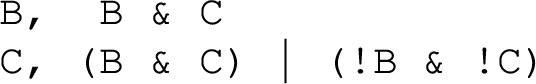


The reduced Boolean network has a new stable motif $\texttt {B}=0$, which defines a minimal trap space $0*$. There no longer is a motif-avoidant attractor.

The reduced Boolean network’s interaction graph is complete. Only by linear extension of all 4 edges would the interaction graph become L-cuttable. Thus, Example 8 illustrates that L-cuttability is not a necessary condition for the absence of a motif-avoidant attractor. Furthermore, the interaction graph of the reduced system satisfies the three criteria identified by Richard and Tonello (2023) as necessary for a Boolean network to be non-separating. Yet, the system is in fact separating: there is a point attractor 11 and a complex attractor that fills the $0*$ subspace. The lack of motif avoidance in the reduced system suggests that the currently known criteria for identifying it are too strict.

The reduced system of Example 8 also illustrates that an attractor that has a single oscillating variable and has fixed states for all other nodes corresponds to a minimal trap space. Two variables must oscillate for a motif-avoidant attractor to exist.

In the examples we encountered, we observed that the MAA-trapping stable motif emerges due to the elimination of a variable’s dependence on a regulator. This happens because newly coincident influences yield a constant due to a logical identity (such as X | !X $\equiv$ 1).

### Reduction introduces motif-avoidant attractors to biological networks

Knowing that node deletions eliminate L-cuttability and generally increase network density, our goal is now to test whether node deletions increase the MAA likelihood in biological networks. However, as we showed with the previous examples, node deletions can both create and destroy MAAs. To reflect these possibilities, we set up our numerical experiment as follows:

For each model from the BBM dataset, we test all input valuations if there are fewer than seven inputs or sample 128 unique constant input valuations. This process results in 14 136 model variants. Then, for each of these model variants, we generate reduced Boolean networks by eliminating variables one by one until no further elimination is possible (i.e. each remaining variable has a self-regulation). To select the variable for elimination, we prioritize those with simple update functions (i.e. small binary decision diagram representations). We then output each such network as a separate test instance, resulting in 1 157 940 Boolean networks. Out of these models, we were able to analyze $\sim$ 750 000 within a timeout of 10 minutes per model. In particular, the results account for all models with fewer than 78 nodes. Based on Sect. 5.2, we would expect MAAs to be increasingly rare in larger models, which is why we have not pursued a longer timeout.

Among these Boolean networks, we identified 9 MAAs with between 4 and 10 oscillating variables. When mapping these nine instances to the original, non-reduced models, they correspond to two models: BBM-151, originally published in Sánchez-Villanueva et al. (2019) and BBM-202, originally published in Gupta et al. (2022). Later in Sect. 8, we provide a detailed discussion of what causes these MAAs to appear in these models and explore their biological relevance.

We should also note that while we made a substantial effort to consider a wide range of model configurations and their reductions, we did use a deterministic method to select which variable is reduced. The order in which variables are reduced does matter in many cases. For example, after identifying the root cause of motif avoidance in models BBM-151 and BBM-202, we could manually derive other reduced variants of these models (often by applying fewer reductions) that admit MAAs. As such, our results cannot be considered exhaustive. Nevertheless, the total number of possible reductions across all BBM models is astronomical, so with this numerical experiment, we hope to provide an illustration of how rare motif-avoidant attractors are even in reduced networks. The emergence of these motif-avoidant attractors motivates further study into the relationship between node deletion and motif avoidance.

### Reduction introduces motif-avoidant attractors to sparse random networks

**Fig. 3:**
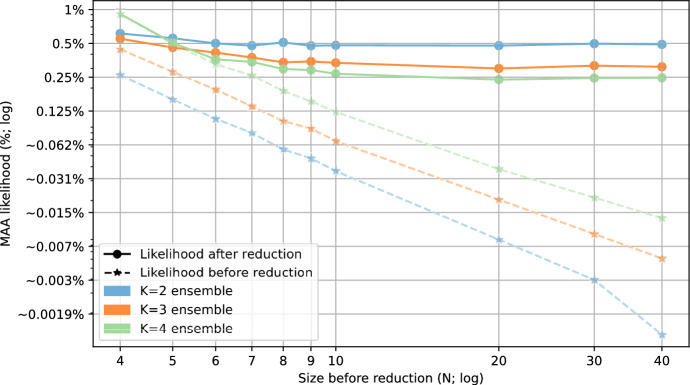
The likelihood of encountering a motif-avoidant attractor *after* maximally reducing the networks (solid trend-line) from the random critical N-K ensembles (see Table B1) compared to the likelihood of the full networks before reduction (dashed trend-lines). The 95% confidence intervals (Wilson score intervals) for all points are smaller than the displayed points. Note that both axes are logarithmic

As we have seen, node deletion can, in practice, lead to the introduction of MAAs. To quantify this effect, we again turn to the critical Random Boolean Networks. We take the random networks sampled in Sect. 5.2 and we reduce each network according to the process outlined in Sect. 6.2. However, in this case, we are only interested in the final, maximally reduced Boolean network, where no further reductions are possible. We then test each such maximally reduced network for MAAs and record the results, just as in Sect. 5.2. We stop once 1000 fully reduced Boolean networks with motif avoidance are observed in each ensemble.

Based on this data, we can compare the likelihood of encountering an MAA in a sampled random Boolean network to the MAA likelihood if said network were to be fully reduced. This trend is visualized in Fig. 3 (the case of $K=5$ was measured, but is omitted for visual clarity). We see that in a set of original (non-reduced) networks with a fixed value of *K*, the MAA likelihood decreases approximately exponentially with *N*. However, if these networks are fully reduced, their MAA likelihood converges to a constant value, and does not decrease meaningfully even for relatively large networks (i.e., for N = 40).

This result could be partially attributed to the fact that in sparse networks, the node deletion reduction is extremely effective at decreasing the overall network size: In the case of $K=2$, the average size of the reduced network is 2.0 for an original network with N = 10 and 2.7 for N = 40. However, if these reduced networks were random, even such a small difference in size (2.0 versus 2.7) should result in a meaningfully different MAA likelihood (see Fig. 2). Yet, the observed likelihood is $0.48\%$ in both cases. For larger *K*, the differences in size are even greater: For $K=5$, we have an average fully reduced size of 5.9 for N = 20 and 8.9 for N = 40, yet the likelihood is the same at $0.22\%$.

We observe that the average number of interactions per variable in these reduced networks also increases with *N* (and thus with the degree of network reduction): In our first example, it goes from 1.25 to 1.43, while in the second example, it increases from 4.5 to 6.9 interactions per variable. This suggests that the MAA likelihood for the fully reduced networks is kept constant by the interplay between network size and the number of interactions. Even though larger networks cannot be reduced as much as the smaller ones, the number of interactions per variable in the reduced networks of larger size is (perhaps counter-intuitively) also greater. Together, our results suggest that these two effects cancel each other out exactly (larger networks have a smaller MAA likelihood, but more interactions increase the MAA likelihood), resulting in the MAA likelihood staying constant.

Our analysis demonstrates that even though motif-avoidant attractors are extremely rare in both biological and random networks, network reduction does meaningfully alter the likelihood of their presence. Furthermore, at least for critical random networks with a fixed *K*, the MAA likelihood of a maximally reduced network does not actually decrease with the original *N*. Modelers always face the question of what resolution to use when constructing a model of a biological process. Should every gene product be included in the model, or can linear pathways be shortened into edges? Our results caution against the indiscriminate use of compression during modeling.

## Investigating the robustness of motif avoidance to delays

After having documented the increase of the MAA likelihood caused by network reduction, we turn to the opposite process: network expansion by linear extension of edges. Naldi et al. (2023) identified a contributing factor to motif avoidance in the unreachability of certain states under asynchronous update due to unsatisfiable requirements on the order of update of the nodes. Although L-cuttability (achieved by linear extension of up to all edges in the network) guarantees the lack of MAAs, we found that MAAs are rare even in non-L-cuttable networks. This finding leads us to propose that motif avoidance can be eliminated by fewer linear extensions than those required by L-cuttability. We evaluate this proposition by investigating two limiting cases. We identify (theoretically and numerically on the MAAs in our collection) cases when a motif-avoidant attractor is eliminated by linear extension of a single edge. We also present leads toward lowering the upper bound for the number of delays needed to eliminate an MAA.

The basis of the elimination of a motif-avoidant attractor is the property that any state that is outside a trap space but can reach the trap space cannot be part of an attractor. Thus, if linear extension of a single edge (which adds a delay variable and doubles the number of states in the Boolean network) makes the states derived from the original system’s MAA reach the trap space, the delay-expanded system no longer has an MAA. Linear extension of an edge is the reverse of the deletion reduction of a linear node. Thus, we can apply the relationships between the trajectories of a reduced Boolean network obtained by deleting a self-edge-free node and the trajectories of the original network (Naldi et al. 2011) to derive reachability relationships in a system in which a single edge is extended by a linear node. In this context, we call the states referred to as ‘representative states’ by Naldi et al. ‘canonical states’.

Paraphrasing Lemma 1 of Naldi et al. (2011) yields information on transitions that start from canonical states. For every state transition $S_1\rightarrow S_2$ in the original system, there will be a transition starting from the canonical state corresponding to $S_1$ in the delay-expanded system and reaching a state corresponding to $S_2$ in the delay-expanded system (canonical or non-canonical). Since the non-canonical states (memory states) can always reach the corresponding canonical state by updating the delay node, this means that the canonical states retain the connectivity of the original system.

Property 1 of Naldi et al. (2011) shows that the delay-expanded system has a transition for which there is no corresponding transition in the original system if and only if (i) the transition starts from a memory state, and (ii) the transition changes the value of the delay-affected variable in a way that is different from the corresponding canonical state. Each state of the delay-expanded system that reaches the trap space due to one of these gained transitions cannot be part of an attractor. If a canonical state derived from a state of the original system’s MAA has a path to the trap space due to one of these gained transitions, the delay-expanded system no longer has an MAA.

A variable affected by a delay can have its update function evaluated to more than one value for a given configuration of the original system, depending on the value of the delay node. As a consequence, memory states can have transitions that the original system did not have (as described above), or can lack transitions that the original system had. We found that lost transitions can interfere with the ability of gained transitions to eliminate the motif-avoidant attractor (see Figure A1 for example). Unlike the gained transitions of memory states, lost transitions of memory states have not been previously studied (to our knowledge). We present a brief overview of lost transitions in Appendix A.

The results concerning the connectivity of canonical states can be generalized from a single to multiple linear extensions. The canonical states of a delay-expanded system can have more connectivity compared to the original system (due to the transitions of memory states that are different from the transitions of the original system), but never less. Furthermore, a Boolean system with multiple delays always retains any connectivity of the canonical states that exist in the Boolean systems that have a subset of those delays.

We use these properties to visualize the new transitions enabled by linear extensions through an economical projection of state transition graphs.

### The projected state transition graph

**Fig. 4:**
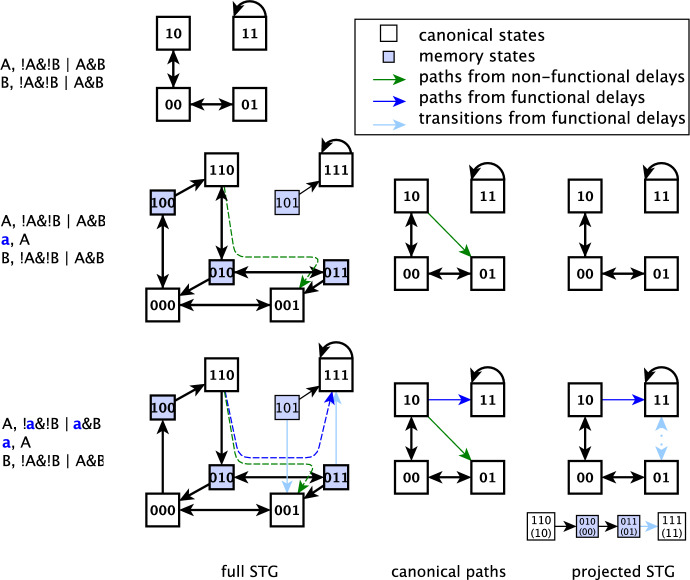
Illustration of linear extensions and projected state transition graphs (STGs) in case of Example 2. Each panel indicates the functions and STG of the original system (top), a system with a non-functional delay node (middle), and a system with linear extension on the self-edge of $\texttt {A}$ (bottom). The states and state transitions that make up the attractor of the original system are shown in bold. Memory states, in which the state of the delay node $\texttt {a}$ is different from the state of its parent node $\texttt {A}$, are marked by a blue background. The light blue edges of the STG show transitions that occur due to the functional delay node. A path in the full STG starting from a canonical state, passing through one or more memory states, and ending in another canonical state is shown in dashed lines, and is projected to an edge among the original system’s states in the second column. Paths that appear in the system with the non-functional delay node are shown with green, and paths that appear only in the system with functional delay are shown with blue. The projected STG adds to the STG of the original system the projections of the new paths allowed by the functional delay node (i.e., the blue edges). Each succession of state transitions that corresponds to a blue edge of the projected STG is indicated below the projected STG; it indicates the corresponding state of the delay-free system in parenthesis and highlights memory states with a light blue background. The projections of the transitions due to the functional delay node (i.e. the projections of the light blue edges) are shown with light blue dotted lines in the projected STG

To effectively illustrate the mechanism by which a linear extension eliminates a motif-avoidant attractor, we propose a mapping of the delay-extended system’s state transition graph to the original system’s state transition graph (STG). We call this mapping the projected state transition graph. In the projected STG a pair of states of the original system is connected by a directed edge if the two corresponding canonical states of the delayed system are connected by a path that does not involve any other canonical states. Note that if a sink node with a single regulator is added to the system, it will create paths among canonical states that do not contain any other canonical states. However, we do not include such paths as edges of the projected STG to focus on the functional effects of the delay node. This choice is similar to the choice of not including in a STG the self-transitions that correspond to a node being updated but not changing state. Fig. 4 illustrates the method of deriving the projected STG of Example 2. The bottom row of Fig. 4 also offers two examples of transitions starting from memory states that are not present in the original system nor for canonical states: $101 \rightarrow 001$ and $011 \rightarrow 111$. We indicate such transitions with blue lines in the delay-expanded system’s STG and with light blue dotted lines in the projected STG.

### Motif-avoidant attractors in small, dense networks can be destroyed by single delays

**Fig. 5:**
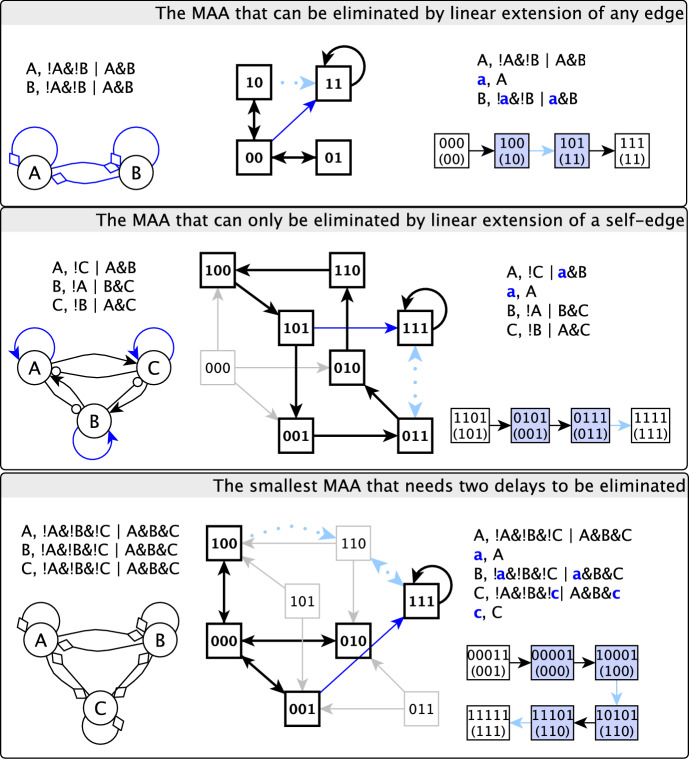
Three prototypical MAAs with minimal connectivity (i.e. eliminating any state transition among states of the MAA would disrupt it) and a trap space 111. In the interaction graph a terminal arrow indicates a positive edge, a terminal circle indicates a negative edge and a terminal diamond indicates a dual edge. For the two MAAs that are possible to eliminate by a single linear extension, the corresponding edges are shown in blue in the interaction graph. For each MAA we indicate the projected STG for one example of a delay set that eliminates the MAA

In our analysis of the motif-avoidant attractors of small, dense networks, we found that single delay nodes (i.e., linear extensions of single edges) were successful in eliminating the MAA. Indeed, a delay to any of the four edges of the 2-variable system of Example 2 will destroy the MAA. In the system of Example 3, linear extension to any of four edges, i.e. $\texttt {A}$ to $\texttt {B}$, $\texttt {A}$ to $\texttt {C}$, $\texttt {B}$ to $\texttt {A}$, $\texttt {B}$ to $\texttt {C}$, will eliminate the MAA. In the system of Example 4, linear extension to the self-regulation of any of the three variables will destroy the MAA. The MAA of Example 5 is destroyed by linear extension of the edge from $\texttt {A}$ to $\texttt {C}$. The motif-avoidant attractor of Example 8 can be eliminated by linear extension to any of four edges, namely the self-regulation of $\texttt {B}$, the self-regulation of $\texttt {C}$, $\texttt {B}$ to $\texttt {C}$ or $\texttt {C}$ to $\texttt {B}$. Furthermore, for 5 of the 6 prototypical 3-variable MAAs, there exist edges whose linear extension eliminates the MAA, as shown in Figure B3 in the Appendix. In Fig. 5 we illustrate three representative systems. Furthermore, we found that all 9 MAAs that come from reduced versions of BBM-202 and BBM-151 can be eliminated by single delays. Figure 7 depicts two such examples of reduced models.

### Motif-avoidant attractors in small RBNs are fragile to delays

**Fig. 6:**
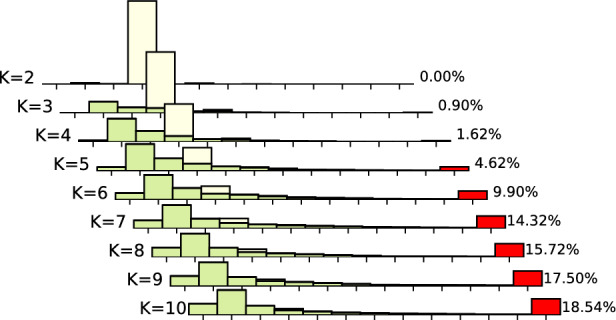
Histogram of the number of interactions among variables oscillating in an MAA wherein inserting a single delay (linear extension) destroys the motif avoidance for critical RBNs with $N=10$ and *K* between 2 and 10. Yellow bars represent MAAs that incorporate the two-variable MAA pattern shown in Example 2. Green bars are all other MAAs. Red bars and the percentages written next to them indicate cases where no single delay destroys the MAA

To better understand the effect single-node delays have on motif avoidance, we return to the critical Random Boolean Networks, and select the $N=10$ ensemble across the full range of *K* values. For each motif-avoidant attractor that was discovered in these networks, we compute the variables that oscillate in this attractor. We then introduce a delay node to each incoming edge of these oscillating variables (each delay is considered separately) and test whether motif avoidance was eliminated.^5^

The results of this analysis are presented in Fig. 6, showing how the number of effective single-delay extensions changes with *K*. The percentage of MAAs that cannot be destroyed by a single delay is very small for $K\le 5$ and even for $K=10$ it is less than $20\%$. We observe that for $K \le 4$, the majority of MAAs can be destroyed by one of four linear extensions (for $K=2$ and $K=3$, this is almost all MAAs). We suspect that this is due to the two-variable MAA of Example 2, which is indeed eliminated by extension of any of its four interactions (see also Fig. 5, middle panel). In sparse networks, it seems unlikely that an MAA appears randomly due to some other combination of interactions. To test this, we further examine the state transition graph of each MAA, detect whether the four-state sub-graph corresponding to the two-variable MAA can be embedded into the detected MAA, and mark these cases with a lighter color in Fig. 6.

For $K=4$ a secondary peak appears, indicating MAAs that can be eliminated by two separate single linear extensions. The simplest example of such MAA is a 3-variable system in which a 2-variable MAA of Example 2 is embedded in the subspace in which the third variable is fixed in the state 0. We describe this example below and show it in Figure B2 in the Appendix.

#### [Style3 Style3]Example 9

(Motif-avoidant attractor destroyable by two alternative single delays) The system below has a trap space 111 and a 2-variable motif-avoidant attractor embedded in the $$**0$$ subspace.
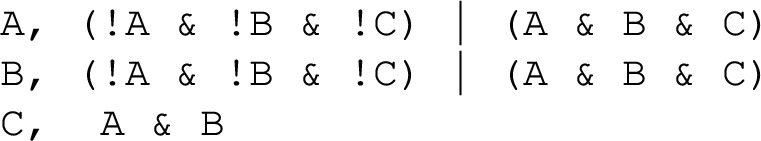


Linear extension of the edge from $\texttt {A}$ to $\texttt {B}$ or of the edge from $\texttt {B}$ to $\texttt {A}$ eliminates the motif-avoidant attractor by creating a path to the canonical state 110, which can reach the trap space.

In conclusion, we observed the fragility of motif-avoidant attractors derived from random networks to linear extensions of single edges. MAAs of sparse networks are especially vulnerable, largely due to the over-representation of the 2-variable MAA of Example  2, which possesses maximal fragility, as extension of any of its edges eliminates motif avoidance. Positioning this motif-avoidant attractor inside a subspace that does not contain the minimal trap space (Example 9) reduces, but does not eliminate, its fragility.

### Efforts to identify an upper bound for the number of delays needed to eliminate a motif-avoidant attractor

Because L-cuttability is sufficient for the non-existence of motif-avoidant attractors, it follows that adding delay nodes to all edges in an interaction graph will eliminate any MAA. Our analyses, however, have shown that in practice substantially fewer delays are needed. We therefore sought to lower the upper bound on the number of delays required to eliminate MAAs by leveraging dynamical information. We identified the size of the subspace that contains the MAA, the Hamming distance between the MAA and the avoided trap space, and the number of variables that have the same function as contributors to this upper bound. We started with simple and plausible conjectures (e.g., that the number of delays is bounded by the Hamming distance between the MAA and the trap space), but found counterexamples for all the conjectures we formulated. Finally, we identified an algorithm that is guaranteed to construct a path of state transitions from a state in the MAA to the trap space using delays on the regulations that drive the system toward the trap space. This algorithm yields an upper bound on the number of delays: $(N-d)(N-1) + m(m+1)/2$, where *N* is the number of variables, $d\le N$ is the number of nodes that oscillate in the MAA, and $m<d$ represents the minimal disagreement between a state of the MAA with (the fixed nodes of) the trap space, when considering only the oscillating nodes of the MAA. In the worst case, with $m=d-1$ and a complete interaction graph with $E_{max}=N^2$ edges, the upper limit is $E_{max}-(\sqrt{E_{max}}-d/2)(d+1)$, which is strictly smaller than $E_{max}$.

The validity of the algorithm automatically proves that the aforementioned upper bound is true. The algorithm utilizes the fact that the subspace of the MAA is not a trap space, and hence, there exists an escape transition out of it. We align a variable of the MAA with the trap space by making this variable have access to the escape transition. This is done by adding delays on its incoming edges from the oscillating variables of the MAA, allowing its update function to read the needed state of the subspace. We indicate the full description of the algorithm and derivation of the number of delays in Appendix A.1. Figure B5 in the Appendix presents an example of applying the algorithm to a system with $N=4$, $d=3$ and $m=1$.

If $N=d$ and $m=1$, the upper bound is 1, meaning that the motif-avoidant attractor can be eliminated by a single linear extension. Application of the algorithm indicates that this linear extension is to the self-regulation of the variable whose state differs in the MAA compared to the trap space. Six of the seven prototypical MAAs shown in Figure B3 have $N=d$ and $m=1$ and indeed can be eliminated by a delay to a single self-regulation.

While the most extensive MAA corresponds to the case in which the smallest trap space that contains the MAA and the avoided minimal trap space is the whole space, another possibility is that this smallest trap space has $M < N$ variables. In this case, the upper bound becomes $(M-d)(M-1) + m(m+1)/2$. A single linear extension eliminates the MAA if $M=d$ and $m=1$. This situation occurs more frequently than the $N=d$ situation described earlier. Indeed, Fig. 6 indicates the high likelihood that Boolean networks with $N=10$ variables contain the 2-variable MAA, which is eliminated by a single delay.

**Fig. 7:**
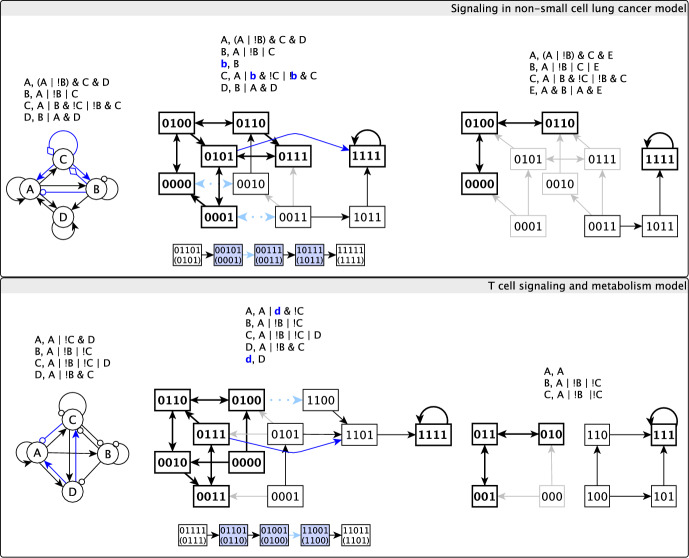
Two motif-avoidant attractors that come from reduced versions of Boolean models. For each system, we indicate the interaction graph and the Boolean functions. Multiple alternative single delays can eliminate each system’s motif-avoidant attractor. We indicate the edges whose individual linear extension can eliminate the MAA in blue and we give the projected state transition graph corresponding to one such example for each network. Both model-derived MAAs illustrate that escaping the subspace of the MAA can be accomplished by linear extension of a subset of the edges incident on the variables fixed by the MAA. For visual clarity, the projected STGs show 10 or 11 states instead of 16. For each system we also include an overlapping or reduced version that has an oscillation contained in a minimal trap space instead of a motif-avoidant attractor

Many of our example MAAs and model-derived MAAs illustrate ways in which the upper bound overestimates the needed number of delays (see Fig. 7 and Figure B2 in the Appendix). The algorithm above relies on the regulations that are part of the stable motif (whose target nodes, when updated, lead to the self-transitions of the states of the trap space). In case of a large Hamming distance between the motif-avoidant attractor and the trap space, using delays to edges that involve state transitions within the MAA offers a potentially better alternative. Indeed, we have identified a family of MAAs that can utilize this aspect. The number of delays necessary and sufficient to eliminate this MAA family, ($\sim E_{max}/4$), is lower than the upper bound. Hence this number represents a lower bound of the number of delays needed in a general algorithm that can eliminate any MAA.

#### [Style3 Style3]Example 10

(The number of delays necessary and sufficient to eliminate this motif-avoidant attractor family scales linearly with $E_{max}$)
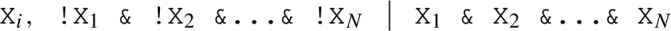


This family of systems has a point attractor 11..1 and a motif-avoidant attractor with $N+1$ states, including 00..0 and all states in which one variable is 1 and the rest are 0. The 2-variable and 3-variable members of this family are illustrated in Figure B3. Because of the star-like shape of the motif-avoidant attractor, we will refer to this family of systems as the star-shaped MAA. Note that we will always consider this MAA in combination with the trap space 11..1. The interaction network of this system is complete, with $E_{max}=N^2$ edges.

According to the upper bound found above, the elimination of this star-shaped MAA should require $N(N-1)/2$ delays. However, the $N=3$ member of this family needs delays to only two edges (instead of 3) to be destroyed. Figure 5 (bottom row) illustrates one possible delay combination. The smaller number of delays is made possible by utilizing a transition within the MAA, in this case the transition from 000 to 010.

Similarly, the $N=4$ member of the family requires 4 delays instead of 6 (compare the second and third panels of Figure B5 in the Appendix). We determined that in the general case the number of delays necessary and sufficient for the elimination of the MAA is $N^2/4$ for even *N* and $(N^2-1)/4$ for odd *N* (see Appendix A.2 for the derivation). Thus, this example has a quadratically scaling number of necessary delays, as the upper bound, but the number of delays is less than (about half of) the upper bound.

In general, having the choice of adding delays such that a transition within the MAA (as an alternative to a self-transition within the trap space) is utilized to align a variable with the trap space is expected to reduce the number of delays needed to eliminate the MAA. In both cases the transition starts from a memory state of the delay-expanded system. In order for this transition to eliminate the MAA, this memory state needs to be reachable from the canonical states derived from the original system’s MAA. We discovered that the general reachability of memory states is not guaranteed. The reason for the loss of reachability is the loss of transitions in certain memory states compared to the original system (and to canonical states). When a memory state affected by such loss becomes a Garden of Eden state, it and potentially additional memory states become unreachable from the MAA. In Appendix A.3 we indicate an example of a delay failing to eliminate the MAA of Example 4 due to an unreachable memory state and contrast it to the effectiveness of a delay affecting a self-regulation. Our algorithm for the upper bound of the number of delays is designed such that it utilizes only the reachable memory states. The star-shaped MAA family avoids unreachability issues due to their unique shape. While the unreachability issue may not be avoidable in general, we did not find any examples in which it caused the lack of all alternatives to destroy an MAA. We indicate more details in Appendix A.3.

We finish this section by turning back from worst-case scenarios of MAA elimination to empirically observed elimination of MAAs.

## The motif-avoidant attractors in reduced real-world models are non-biological and easily eliminated or trapped

Among the 230 BBM models, we found two models whose reduced versions exhibited motif-avoidant attractors. The first model (Gupta et al. 2022) describes signal transduction in non-small cell lung cancer. It has 28 nodes, including the input node “$\texttt {DNA-damage}$”, and 107 edges. The original article focuses on the setting $\texttt {DNA-damage}=1$ and reports two point attractors, describing cell cycle arrest and apoptosis, respectively. The setting $\texttt {DNA-damage}=0$ corresponds to cancer cell proliferation. The MAA only appears in this latter setting, in reduced versions of the model with 4 to 8 nodes. Its appearance is enabled by an unintended feature of the update function of $\texttt {p53-A}$, a modified form of the tumor suppressor protein $\texttt {p53}$, which allows the activity of $\texttt {p53-A}$ even if the parent $\texttt {p53}$ protein is inactive. This outcome of the function is inconsequential in the setting of $\texttt {DNA-damage}=1$, thus it likely avoided the attention of the modelers.

We illustrate a four-node reduced version of the model in Fig. 7. For visual clarity, we shortened the variable names and performed a parity change in three of the variables. Node $$\texttt {A}$$ of this system corresponds to the activity of the protein $$\texttt {c-Myc}$$ in the original model, node $$\texttt {B}$$ represents the inactivity of $$\texttt {p53}$$, node $$\texttt {C}$$ represents the inactivity of $$\texttt {p53-A}$$, and node $$\texttt {D}$$ represents the inactivity of the microRNA $$\texttt {miR-34a}$$. The system has a trap space $$1*11$$, a minimal trap space (point attractor) 1111, as well as an MAA that visits 6 states of the $$0***$$ subspace. The point attractor agrees with a proliferative phenotype, while the MAA cannot be attributed biological meaning. The state 0011 has a transition to 1011, thus the $$0***$$ subspace is not a trap space. The MAA can be eliminated by a linear extension of a single edge; the five edges that are successful candidates are indicated by blue color in the interaction graph. We also provide the projected STG for one of these extensions. Alternative reductions of the model feature a complex attractor that is inside a minimal trap space. We illustrate one such model, which overlaps the previous example in three nodes and has the additional node $$\texttt {E}$$, which represents the cell cycle protein $$\texttt {YY1}$$. This system has a minimal trap space 1111 as well as a 3-state oscillation within the $$0**0$$ trap space.

The second model (Sánchez-Villanueva et al. 2019) connects metabolism, the production of reactive oxygen species ($\texttt {ROS}$), and T cell signaling. It contains 111 nodes, including two input nodes, the T cell receptor $\texttt {TCR}$ and the co-stimulatory protein $\texttt {CD28}$, and 244 edges. In the original model 21 nodes have 3 states (0, 1, 2), with separate functions for the level 1 and the level 2. The model was made Boolean by representing the two levels as two separate nodes. The original study reports that in the absence of $\texttt {TCR}$ or $\texttt {CD28}$, cells remain quiescent or are kept in metabolic anergy (inactivity). In the presence of both $\texttt {TCR}$ and $\texttt {CD28}$ signals, cells could either adopt an anergic state with high $\texttt {ROS}$, or undergo activation. However, this model includes a hidden constant that is introduced by the Booleanization process: the function that describes level 1 of node $\texttt {ETC}$, which represents the enzymes involved in the electron transport chain, always evaluates to 1. Our method interpreted this constant as a source node, and found MAAs in the setting where this constant level is set to 0. Biologically, this corresponds to a perturbation of the original system that blocks the activity of the electron transport chain, coupled with the inactivity of either $\texttt {TCR}$ or $\texttt {CD28}$. In other words, the conditions captured by the MAA do not represent a biological setting originally considered when designing the model, but rather a perturbation whose biological realizability has not been tested.

We illustrate a four-node reduced version of the model in Fig. 7. Node $$\texttt {A}$$ represents a high level of mitochondrial $$\texttt {ROS}$$, node $$\texttt {B}$$ represents the inactivity or the ATP synthase enzyme, node $$\texttt {C}$$ represents a lack of thioredoxin pool in the mitochondria, and node $$\texttt {D}$$ represents a high activity of the enzyme thioredoxin reductase. This system has a stable motif $$\texttt {A}=1$$, a minimal trap space 1111, as well as an MAA that visits 5 states of the $$0***$$ subspace. The point attractor corresponds to an anergic state with high $$\texttt {ROS}$$, while the MAA does not have a known biological interpretation. The motif-avoidant attractor can be eliminated by a single delay to any of three edges (shown in blue in the interaction graph). This model can be simplified further by reducing the node $$\texttt {D}$$. The 3-node system has a minimal trap space 111 and instead of the MAA has a 3-state oscillation contained in the minimal trap space $$0**$$. The reason for this change in the nature of the oscillation is the emergence of a new stable motif, $$\texttt {A}=0$$. This stable motif arises because upon substituting the function of $$\texttt {D}$$ into the function of $$\texttt {A}$$, the second clause becomes "!C & !B & C", which evaluates to 0.

In summary, the motif-avoidant attractors in both models are unintended and appear only for specific input values and specific instances of model compression. Other choices of model compression lead to trapped oscillations or no oscillatory attractor. The MAAs are prone to elimination by single linear extension and prone to trapping by node reduction.

## Discussion

Boolean networks are fruitful models of biological systems because their attractors correspond to biological phenotypes, and can identify which initial conditions lead to which outcomes (Albert et al. 2008), which components contribute to certain phenotypes (Beneš et al. 2022), and which interventions can drive the system toward a target behavior (Paul et al. 2019; Cifuentes-Fontanals et al. 2022; Brim et al. 2023). There are various efficient methods to identify the attractors of Boolean systems (Mori and Akutsu 2022; Rozum et al. 2021; Beneš et al. 2021; Van Giang and Hiraishi 2021; Trinh et al. 2024). Yet, motif avoidance poses a significant obstacle to these methods, and the search for motif-avoidant attractors is computationally difficult (Rozum et al. 2021).

Motif avoidance plays an important role in synchronous update and underlies the cases in which a sustained oscillation arises from a positive feedback loop (as opposed to a negative feedback loop). Synchronous MAAs are a significant contributor to the substantially higher number of attractors in Random Boolean Networks (RBNs) under synchronous update (Kaufman et al. 2005) compared to asynchronous update (Greil and Drossel 2005; Rozum et al. 2021). Multiple Boolean models of biological systems that yield sustained oscillations rely on motif avoidance under synchronous update (Park et al. 2023; Dahlhaus et al. 2016; Orlando et al. 2008). Yet, the frequency of motif-avoidant attractors under asynchronous update has not been systematically studied before.

To fill this knowledge gap, we performed an extensive search in 230 published Boolean models with 14 136 input combinations and more than 100 million RBNs. We found that MAAs are very rare. There were no MAAs in the published Boolean models. A small percentage of small and dense RBNs have motif-avoidant attractors ($\sim \!\! 1.6\%$ for complete networks of 10 nodes) and in large, sparse RBNs the MAA likelihood is astronomically small.

Although the conclusion that modelers of biological systems don’t need to concern themselves with motif-avoidance is inviting, the reality is more subtle. Our numerical analysis of reduced versions of the Boolean models and maximally reduced versions of RBNs indicates that network reduction increases the MAA likelihood. The MAA likelihood in maximally reduced RBNs is similar to the MAA likelihood of small and dense networks. Furthermore, we identified 9 MAAs in reduced versions of two published Boolean models. We could not ascribe biological relevance to either MAA. Our results suggest that compression of information during model construction may inadvertently lead to the emergence of MAAs via surprising non-local network effects. As such compression of information is unavoidable and increasingly automated, the possibility of motif avoidance should not be ignored by modelers.

We performed computational and theoretical studies of MAAs, whose results, summarized in Table 1, yield a better understanding of the mechanisms that contribute to motif avoidance. We identified small networks that give rise to prototypical MAAs with minimal state transitions. We showed that they (and in particular the 2-variable MAA presented in Example 2) play a major determinative role in the presence of MAAs in both random and reduced biological networks. We analyzed the role of canalization in these MAA-containing networks, finding that low canalization favors MAAs. Future studies of the canalization properties of systems with MAAs with non-minimal connectivity in the state transition graph may find that such systems are even less canalizing.

By analyzing our ensemble of MAAs arising in RBNs, we found that they are fragile to linear extension of single edges. We have proved that an MAA in the same subspace as an avoided trap space can always be eliminated by a single linear extension if the minimal Hamming distance between the MAA and the trap space is one. Indeed, 6 of the 7 small, dense Boolean networks that exhibit prototypical MAAs have this property. The MAAs that arise from reduced versions of Boolean models of biological systems, though they are more complex (they have more variables and are not in the same subspace as the avoided trap space), can also be eliminated by single linear extensions. These results pose an interesting contrast to the currently known upper bound for the number of delays needed to eliminate a motif-avoidant attractor, namely, that linear extensions of up to all the edges are needed such that the interaction graph becomes L-cuttable. We lowered the upper bound using limited dynamic information, and showed that this bound cannot be lowered below $\lfloor N^2/4 \rfloor$. Our algorithms used to derive the upper bound can be used together with network decomposition or subspace restriction techniques to reduce the upper bound in specific cases.

The currently known theoretical criteria for ruling out MAAs based on the interaction network (Naldi et al. 2023; Richard and Tonello 2023) are strict. We noted that only 9 of the 230 published Boolean models have an L-cuttable interaction graph. Yet, we also noted that when considering fixed values of the input nodes (which denote external signals or cellular context), and percolating these values through the model, $38\%$ of the instances yield an empty network (indicative of monostability for each input configuration), and $9\%$ of the instances yield an L-cuttable network. This simplification of the “effective” interaction graph based on dynamic context may contribute to the lack of MAAs in biological networks and warrants further study, for example using the methods of Gates et al. (2021).

Significant open questions remain. For example, at present, there is no estimate of the prevalence of the unreachability issue that limits the candidates for linear extensions to regulations of the trap space. We discuss potential leads in Appendix A.3. We expect that the upper bound for the number of delays necessary to eliminate an MAA can be lowered in the classes of motif-avoidant attractors in which this issue can be ruled out; this may include all MAAs.

We propose that an interesting research venue to pursue by the community of mathematical biologists is the identification of large subclasses of Boolean networks that don’t have motif-avoidant attractors. From previous theoretical work (Richard and Comet 2007; Richard 2010; Richard and Tonello 2023; Naldi et al. 2023) we know that Boolean networks whose interaction graph has no cycles, has only positive or only negative cycles, lacks a path from a positive cycle to a negative cycle, or is L-cuttable, cannot have MAAs. These criteria leave many possibilities, especially in dense networks, in which motif avoidance cannot be ruled out. As described in Sect. 4, we considered several natural candidates for additional criteria on the structure of the interaction network, or on the Boolean update functions, and found counterexamples for them. The desired large subclass of Boolean networks should exclude the sole 2-variable MAA and the six prototypical 3-variable MAAs. We expect that future work will identify the patterns (subnetworks of Boolean systems) that are necessary for motif avoidance.

Identification of a large MAA-free subclass will significantly aid algorithm design: If MAAs can be ruled out, attractor search becomes much easier in practice. Our work currently offers empirical evidence for designers of tools geared toward biological systems; they can use this evidence to argue whether they need to account for MAAs in their algorithms.

Increased clarity on the minimal number of delays to eliminate a motif-avoidant attractor will help with the biological interpretation of motif avoidance. MAAs under asynchronous or random set update retain an element of synchronicity: the effect of a variable’s state change is immediately reflected in all update functions it regulates. Though this may be a useful approximation in many cases, it fails to account for inherent delays in a system, such as when a protein must slowly diffuse through the cytoplasm to reach its target. Boolean systems with explicit delays associated with each edge are a way of including such non-negligible durations (Cheng et al. 2013; Akman et al. 2023). Identifying which delays affect the long-term dynamics of a Boolean network would be useful in building more biologically realistic models.

A clearer picture of the reasons that motif-avoidant attractors are rare in asynchronous Boolean networks is beginning to emerge. One hint is that the small and dense subnetworks associated with MAAs are rare in biologically relevant Boolean models (whether empirical or random). Another hint is the strong canalizing role played by input nodes in Boolean models of biological systems, which fix large portions of the network, making many network edges functionally redundant. This effectively eliminates any dense subnetworks that do appear and might otherwise give rise to motif avoidance. This is supported by our observation that the functions of prototypical 3-variable MAAs have lower canalization measures than biological models and critical RBNs on average. A third hint is the fragility of MAAs to single delays, which we have shown can arise when MAAs closely approach avoided trap spaces. These hints do not yet give the whole picture, but they may point the way to a more complete understanding and computationally efficient characterization of motif avoidance.

## Supplementary Information

Below is the link to the electronic supplementary material.

Supplementary file 1 (pdf 867 KB)

## Data Availability

The networks, code and raw results upon which we base our analysis are available as part of a reproducibility artefact at https://doi.org/10.5281/zenodo.13860057.

## Footnotes

1 An alternative representation of an ambiguous edge is a superposition of an activating edge and an inhibiting edge.

2 Sampling of the [N = 40, K = 4] and [N = 40, K = 5] ensembles had to be terminated prematurely after several weeks of runtime. The fact that these ensembles contain fewer than 1000 motif-avoidant cases is accounted for in the subsequent analysis.

3 The remaining 22 models are all variants of a single BBM model that can, depending on the input values, exhibit a very large number of minimal trap spaces that could not be fully enumerated even with a one week timeout.

4 Note that these are not the same nine models that could not be analyzed using AEON.py. Instead, these are small or structurally simple models that were easy to analyze exhaustively.

5 In the presence of multiple MAAs in one network, it is not clear how to associate the attractors of the original and the extended systems. In such cases, we conservatively consider the delay to be “working” only if it eliminates all MAAs.
