# Supplementary material for: An open problem: Why are motif-avoidant attractors so rare in asynchronous Boolean networks?
Source: J Math Biol. 2025 Jun 12;91(1):11. doi: 10.1007/s00285-025-02235-8 (PMC12162798; doi:10.1007/s00285-025-02235-8)
Supplement: Supplementary file 1 — (pdf 867 KB) [file 285_2025_2235_MOESM1_ESM.pdf]

Appendix A Supplementary information on the number of delays needed to eliminate a motif-avoidant attractor

A.1 Proof of the validity of the algorithm and upper bound for the number of delays needed to eliminate a motif-avoidant attractor

Consider a Boolean network of N variables that contains a known stable motif and its trap space $\mathcal{X}$ in which n variables are fixed (and $N-n$ are free to vary), and a MAA $\mathcal{A}$ in which d variables oscillate. Without loss of generality, we take the first n variables of the network to have the ON state within $\mathcal{X}$, and also assume that the smallest trap space that contains both $\mathcal{A}$ and $\mathcal{X}$ is the whole state space. When the latter is not true, the algorithm can simply be applied to the M variables that are free to vary in the smallest trap space that contains both $\mathcal{A}$ and $\mathcal{X}$. In the following, we describe an algorithm that can be used to determine the set of delays (linear extensions of edges) which if simultaneously added to the system, will eliminate the MAA.

For simplicity, in the following description we will refer to states (not necessarily unique) of the delay-expanded system by indicating the states of the original (non-delay) variables. The corresponding states in the delay-expanded system are canonical states, or memory states in which each delay node acts as a memory of a past state of its parent node. In our proofs, we describe the addition of delays in an iterative way, but in practice they are all added at once. We distinguish the different iterations using superscripts. For example, system state $S_1^{(1)}$ and system state $S_1^{(2)}$ will have the same state of the original variables, but $S_1^{(2)}$ is a state of the system expanded with more delay nodes.

As the first step, we show that in the worst case, $(N-d)(N-1)$ delays are sufficient to expand the MAA such that it oscillates in all N variables. Consider the $N-d$ variables $\{\mathtt{F}_i\}$ that are fixed to the corresponding values (node states) $\{f_i\}$ in $\mathcal{A}$. $\mathcal{A}$ lies within the $\{\mathtt{F}_i = f_i\}$ subspace and spans the subspace (meaning that all d free variables of the subspace oscillate in $\mathcal{A}$). By adding delays to all the incoming edges of $\{\mathtt{F}_i\}$ (except self-edges), we ensure that each variable $\mathtt{F}_i$ can read any state in the $\{\mathtt{F}_i = f_i\}$ subspace for its update.

This can be shown in an iterative way. Since the subspace that contains $\mathcal{A}$ is not a trap space, one of its states must have a transition that turns a variable among $\{\mathtt{F}_i\}$ to its opposite state. Let's say system state S_1 has a transition that turns variable $\mathtt{F}_1$ to state $!f_1$. Having delay nodes on all incoming edges of $\mathtt{F}_1$ (except the self-edge) allows it to read this state and make the transition. As the delayed system oscillates through the states that correspond to $\mathcal{A}$ (but with new delay nodes), the d variables oscillate, and the delay nodes that correspond to these d variables can freely retain any state. This is possible because the delay nodes only affect $\mathtt{F}_1$, and the transitions within $\mathcal{A}$ do not involve the update of $\mathtt{F}_1$. (If $\mathtt{F}_1$ was updated in a path, the delay nodes would need to be in specific states to allow such a transition.) Thus, it is possible for the delay nodes to be updated to a combination that matches S_1. Note that this allows $\mathtt{F}_1$ to turn to state $!f_1$ in any system state that corresponds to $\mathcal{A}$ (but with

the aforementioned delay node states fixed to the required values). The self-edge is not needed because $\mathtt{F}_1 = f_1$ in any state that corresponds to $\mathcal{A}$, as in S_1. If $\mathtt{F}_1 = \mathtt{!}f_1$ creates a path to the previously avoided trap space, the goal of eliminating the MAA is achieved. Otherwise, the transition will lead back to the MAA, and $\mathtt{F}_1$ will oscillate. Let's call the resultant attractor $\mathcal{A}^{(1)}$.

Next, consider a larger subspace (of the original system) wherein $\mathtt{F}_1$ and the d variables are free to vary. This subspace is not a trap space. If this subspace were a trap space that contains $\mathcal{X}$, it would contradict the assumption that the whole state space is the smallest trap space that contains both $\mathcal{A}$ and $\mathcal{X}$. If this subspace were a trap space that does not contain $\mathcal{X}$, then $\mathcal{A}$ is not an MAA. Again, since this subspace is not a trap space, there must exist a system state in which variable $\mathtt{F}_2$ turns to state $\mathtt{!}f_2$. Adding delays to all the incoming non-self-edges of $\mathtt{F}_2$ allows this transition from any system state that corresponds to $\mathcal{A}^{(1)}$, again because the d variables and $\mathtt{F}_1$ oscillate in $\mathcal{A}^{(1)}$, and the new delays only affect $\mathtt{F}_2$. Similarly, when all d variables and the originally fixed variables up to $\mathtt{F}_k$ are allowed to oscillate in $\mathcal{A}^{(k)}$, having delays on all incoming non-self-edges for variable $\mathtt{F}_{k+1}$ allows it to turn to state $\mathtt{!}f_{k+1}$ from any system state that corresponds to $\mathcal{A}^{(k)}$. By induction, having delay nodes on all the incoming non-self-edges of $\{\mathtt{F}_i\}$ is sufficient to guarantee the transition of all $\{\mathtt{F}_i\}$ variables to the $\{\mathtt{!}f_i\}$ states. In the worst case, each variable $\mathtt{F}_i$ has $N-1$ regulators other than itself, leading to an upper bound of $(N-d)(N-1)$ delays added in this process. Note that at this point, all N original variables oscillate in the MAA $\mathcal{A}^{(n)}$. Furthermore, from any system state that corresponds to $\mathcal{A}$, we can make all the $N-d$ variables $\{\mathtt{F}_i\}$ agree with $\mathcal{X}$ in a sequential manner. However, this still does not guarantee that the avoided trap space can be reached.

Following this, consider the d variables $\{\mathtt{A}_i\}$ that initially oscillate in $\mathcal{A}$. Among the states of $\mathcal{A}$, consider a state S wherein the smallest number of variables, say m, among $\{\mathtt{A}_i\}$ disagree with $\mathcal{X}$. As described above, using at most $(N-d)(N-1)$ delays on the edges incident on $\{\mathtt{F}_i\}$, we can change the state of all the variables $\{\mathtt{F}_i\}$ to agree with $\mathcal{X}$. Let's say this is done by following a path of state transitions $\mathcal{P}$. $\mathcal{P}$ visits system states in which each of $\{\mathtt{A}_i\}$ are ON, states in which each of $\{\mathtt{A}_i\}$ are OFF, leads to state S, then turns variables $\{\mathtt{F}_i\}$ ON, resulting in state S_{n-m}. S_{n-m} has m variables among $\{\mathtt{A}_i\}$ that disagree with $\mathcal{X}$ (i.e., they are in the OFF state), while all variables $\{\mathtt{F}_i\}$ agree with $\mathcal{X}$ (i.e., they are in the ON state). Such a path always exists because the delay nodes incident on each F_i have access to both the ON and OFF state of all of the $\{\mathtt{A}_i\}$ and $\{F_{j<i}\}$ variables. In our later steps, we omit referring to the $(N-d)(N-1)$ delay nodes for simplicity; they are implicitly updated appropriately along $\mathcal{P}$.

In the second step, the task is to turn ON the $m < d$ variables among $\{\mathtt{A}_i\}$ that are OFF in S_{n-m}. Let's call this subset of m variables $\{\mathtt{X}_i\}$. Note that due to the self-transitions of $\mathcal{X}$, each variable $\mathtt{X}_i$ can turn ON if all other variables among the n variables fixed in $\mathcal{X}$ are ON. We utilize this by accessing the history of ON states of these variables previously visited within the attractor.

When the path $\mathcal{P}$ visits a state with variable $\mathtt{X}_j$ ON, the $\mathtt{X}_j =$ ON state can be stored in a delay node that regulates some other variable $\mathtt{X}_i$. Our aim is to make sure that each variable $\mathtt{X}_i$ can have access to the ON states of the delay nodes of its original

regulators (which could include $\mathtt{X}_j$) in system state S_{n-m}, so that it can turn ON. Note that since $\mathtt{X}_i$ is OFF in S_{n-m}, the path leading to S_{n-m} must turn OFF $\mathtt{X}_i$ at least once. However, $\mathtt{X}_i$ may be unable to turn OFF if the state of the incident delay node is ON. For example, if the original update function of $\mathtt{X}_i$ requires the OFF state of $\mathtt{X}_j$ to turn OFF $\mathtt{X}_i$, the delayed system can only turn OFF $\mathtt{X}_i$ when the delay node corresponding to $\mathtt{X}_j \to \mathtt{X}_i$ is OFF. The problem arises because the same delay node needs to be ON to turn ON $\mathtt{X}_i$ in later stages of the path.

This problem can be avoided if we arrange $\{\mathtt{X}_i\}$ in the order of their last ON states, i.e., so that $\mathtt{X}_1$ is the variable whose last ON state is the first among $\mathtt{X}_i$ in the path, $\mathtt{X}_2$ is the variable whose last ON state is the second, and $\mathtt{X}_m$ is the variable whose last ON state is the last in the path. Note that each of the $\mathtt{X}_i$ variables may turn ON multiple times, but we only consider their last ON state. This allows the delay nodes of $\{\mathtt{X}_{j>i}\}$ to be updated to ON *after* $\mathtt{X}_i$ turns OFF. In the case of a self-edge, a delay of $\mathtt{X}_i$ ON does not prevent $\mathtt{X}_i$ from turning OFF. (Since the delay node and the parent node are in the same state, if $\mathtt{X}_i$ could turn OFF in the original system, it can still turn OFF in the delayed system. The delay node can freely retain the ON state of $\mathtt{X}_i$.)

Let $\mathtt{X}_1$ have delays on its edges that start from $\{\mathtt{X}_{i\geq 1}\}$ (including itself if it has a self-edge, at most m). Consider an instance of $\mathcal{P}$, $\mathcal{P}^{(1)}$, which visits a state where $\mathtt{X}_1$ is ON, visits states where $\mathtt{X}_i$ is ON in a sequence without turning $\mathtt{X}_1$ ON again, and reaches system state $S^{(1)}_{n-m}$, while updating the delay nodes incident on $\mathtt{X}_1$ ON. At $S^{(1)}_{n-m}$, all the delay nodes incident on $\mathtt{X}_1$, along with the other $n-m$ non-delay variables, are ON. This allows $\mathtt{X}_1$ to turn ON using the self-transitions in $\mathcal{X}$ (which make the stable motif "stable"). This leads to a system state $S^{(1)}_{n-(m-1)}$ that has $m-1$ nodes among $\{\mathtt{A}_i\}$ that disagree with $\mathcal{X}$. We repeat the process by letting $\mathtt{X}_2$ have delays on its edges that start from $\{\mathtt{X}_{i\geq 2}\}$ (at most $m-1$). A new path $\mathcal{P}^{(2)}$ again visits states where $\mathtt{X}_1$, $\mathtt{X}_2$, and the rest of $\mathtt{X}_i$ are ON in a sequence, just as in $\mathcal{P}^{(1)}$, but it also updates the delay nodes incident on $\mathtt{X}_2$. At $S^{(2)}_{n-m}$, $\mathtt{X}_1$ can be updated to ON same as before to reach $S^{(2)}_{n-(m-1)}$. Here the delay nodes from $\{\mathtt{X}_{i\geq 2}\}$ incident on $\mathtt{X}_2$, $\mathtt{X}_1$ itself, and the other $n-m$ non-delay variables are in their ON states. This allows $\mathtt{X}_2$ to turn ON and lead to state $S^{(2)}_{n-(m-2)}$. Repeating this process leads to $S^{(m)}_n$, which is part of $\mathcal{X}$, using at most $m(m+1)/2$ delays.

As a result, having $(N-d)(N-1)+m(m+1)/2$ delays guarantees the elimination of the MAA. Here m represents the minimal disagreement between a state of the MAA with (the fixed variables of) the trap space, when this disagreement is considered only among the variables $\{A_i\}$. Note that since delay nodes are unnecessary when there is no regulation, the upper bound can be greatly lowered for non-complete networks. When the smallest trap space that contains both $\mathcal{A}$ and $\mathcal{X}$ has M variables that are free to vary, the upper bound is adapted by simply replacing N by M.

The first panel of Figure B5 illustrates the application of the algorithm to eliminate an MAA with $N=4$, $d=n=3$ and $m=1$. Variable $\mathtt{D}$ is in the OFF state in the four states of the MAA. The transition (1 1 1 0) $\to$ (1 1 1 1) is an escape from the $**{*}0$ subspace. Following the first step of the algorithm, adding delay nodes to the edges from $\mathtt{A}$, $\mathtt{B}$, and $\mathtt{C}$ to $\mathtt{D}$ adds 7 new transitions from memory states with $\mathtt{D}=0$ to memory states with $\mathtt{D}=1$. These transitions expand the MAA from 4 states with

$\mathtt{D} = 0$ to 8 states with oscillating D when considering the states of the original system. (When considering all delay nodes, the expansion is to 52 states. This includes all the 32 states derived from the 4 states of the original MAA, as well as 20 additional states with D in the ON state.) As there isn't a path from the expanded MAA to the trap space, more delays are needed in the second step of the algorithm. The state of the expanded MAA with fewest disagreements with the trap space is (01 01 11 1) (or 0011 considering only the original variables), in which the state of B disagrees with the trap space. Adding a delay on the self-regulation of B allows it to turn ON from the memory state (01 011 11 1), which is part of the strongly connected component of the delay-expanded system's STG. The transition creates a path to the trap space, thus there no longer is an MAA.

A.2 Derivation of the number of delays needed to eliminate the motif-avoidant attractor of Example 10

Consider the family of motif-avoidant attractors defined in Example 10, to which we will refer to as the star-shaped MAA. Here we present the details of an algorithm to eliminate star-shaped MAAs using transitions both within the MAA and within the trap space. The number of delays indicated by this algorithm is one half of the upper bound obtained if only using the self-transitions within the trap space (which is the case in the general algorithm). We then prove that this number of delays cannot be reduced further.

For simplicity, in the following description we will refer to states (not necessarily unique) of the delay-expanded system by indicating the states of the original (non-delay) variables. The corresponding states in the delay-expanded system are canonical states, or memory states in which one or more delay nodes act as a memory of a past state of their parent nodes. We will denote these states using as subscript the number of original variables that are ON. In our proofs, we describe the addition of delays in an iterative way, but in practice they are all added at once. We distinguish the different iterations using superscripts. For example, system states $S_1^{(1)}$ and $S_1^{(2)}$ will have the same state of the original variables, but $S_1^{(2)}$ is a state of the system expanded with more delay nodes.

Algorithm to eliminate the star-shaped MAA:

In the star-shaped MAA the state 00..0 can turn ON any variable $\mathtt{X}_{1\leq i\leq N}$. Suppose we start from a state S_1 where $\mathtt{X}_1$ is ON (all other variables are OFF), and we need to turn ON the remaining $N-1$ variables to reach the trap space 11..1.

Claim 1: The star-shaped MAA can be destroyed by placing $N-k+1$ delay nodes incident on each $\mathtt{X}_k$ $(1 < k \leq N)$. That is, $\frac{(N-1)N}{2}$ delays overall.

One can follow the general algorithm for the upper bound of delays (specifically the second part, since all variables already oscillate in the star-shaped MAA) to accomplish this goal and verify the validity of the claim. The delay nodes incident on $\mathtt{X}_k$ serve as a memory of the ON state of their parent nodes $\{\mathtt{X}_{i\geq k}\}$, allowing $\mathtt{X}_k$ to turn ON using a self-transition within the trap space.

Claim 2: The star-shaped MAA can be destroyed by placing $k-1$ delay nodes incident on each $\mathtt{X}_k$ $(1 < k \leq N)$. That is, $\frac{(N-1)N}{2}$ delays overall.

This claim presents a symmetrical counterpart to Claim 1. In Claim 1 we added $N-1$ delays, one on each edge from variables $\{\mathtt{X}_{j\geq 2}\}$ to variable $\mathtt{X}_2$, to turn $\mathtt{X}_2$ ON. Instead, we can achieve the same with a single delay, on the edge from $\mathtt{X}_1$ to $\mathtt{X}_2$. Consider a transition that starts from the system state $S_0^{(2)}$ (which equals the canonical state 00..0), turns $\mathtt{X}_1$ ON, and reaches state $S_1^{(2)}$. Then the delay node is OFF in state $S_1^{(2)}$, which allows $\mathtt{X}_2$ to turn ON, thereby reaching state $S_2^{(2)}$. Similarly, add two delays on the edges from the variables $\mathtt{X}_1$ and $\mathtt{X}_2$ to $\mathtt{X}_3$. As the system transitions from $S_0^{(3)}$ to $S_2^{(3)}$, the delay nodes preserve the memory of the OFF state of $\mathtt{X}_1$ and $\mathtt{X}_2$, allowing $\mathtt{X}_3$ to turn ON at $S_2^{(3)}$ and reach $S_3^{(3)}$. Repeating this process, suppose there exists a path that starts at $S_0^{(k-1)}$, goes through $S_1^{(k-1)}$, $S_2^{(k-1)}$, ... and arrives at state $S_{k-1}^{(k-1)}$ that has $k-1$ original (non-delay) variables, $\mathtt{X}_1$ to $\mathtt{X}_{k-1}$, ON. Adding $k-1$ delay nodes, on the edges from variables $\{\mathtt{X}_{i<k}\}$ to $\mathtt{X}_k$, allows the system expanded with the new delay nodes (which are kept in the OFF state) to follow a path almost identical to the previous; this path visits the states $S_1^{(k)}$, $S_2^{(k)}$, ... , $S_{k-1}^{(k)}$. Then $\mathtt{X}_k$ can be updated to the ON state in system state $S_{k-1}^{(k)}$, reaching $S_k^{(k)}$. By induction, this process can reach the state $S_N^{(N)}$ wherein all original variables are ON, and then update the delay nodes to reach the trap space. In summary, the delay nodes serve as a memory of the OFF state of their parent nodes $\{\mathtt{X}_{i<k}\}$, allowing $\mathtt{X}_k$ to turn ON using a transition within the MAA.

Claim 3: These two delay schemes are compatible and therefore the star-shaped MAA can be destroyed by placing EITHER $k-1$ or $N-k+1$ delays on each $\mathtt{X}_k$. (The minimal number of added delays in this scheme is characterized by Claim 4.)

In the system without delay nodes, $\mathtt{X}_k$ can be updated to the ON state either if all its regulators are OFF, or if all its regulators are ON. In a system with delay nodes, $\mathtt{X}_k$ can be updated to the ON state from the state S_{k-1} if either the delay nodes in the edges from $\{\mathtt{X}_{i\geq k}\}$ are in their ON states, or if the delay nodes in the edges from $\{\mathtt{X}_{i<k}\}$ are in their OFF states. We show that there exists a path that allows either, independent of the choices for delays for other variables $\mathtt{X}_j$.

Suppose that we have a delay-expanded system that has delays on the edges to variables $\{\mathtt{X}_{1<i<k}\}$, in such a way that there exists a path $\mathcal{P}^{(k-1)}$ from $S_0^{(k-1)}$ (where all original variables are OFF) to $S_{k-1}^{(k-1)}$ (where the original variables $\{\mathtt{X}_{i<k}\}$ are ON). Note that in general $S_0^{(k-1)}$ is not a canonical state, and must have appropriate states of the delay nodes on the edges to variables $\{\mathtt{X}_{i<k}\}$ to allow $\mathcal{P}^{(k-1)}$. Therefore, also assume that there exists a path $\mathcal{P'}^{(k-1)}$ that starts at the canonical state 00..0 in the MAA, visits states where each of the variables (starting from $\mathtt{X}_1$ and ending at $\mathtt{X}_N$) is ON individually, while going back to a canonical or memory variant of 00..0 each time, and reaching $S_0^{(k-1)}$. $\mathcal{P}^{(k-1)}$ and $\mathcal{P'}^{(k-1)}$ together ensure that this system has a path from the canonical state 00..0 to a state $S_{k-1}^{(k-1)}$.

Now we add delays to the edges on $\mathtt{X}_k$. If we add $N-k+1$ delays on the edges from $\{\mathtt{X}_{i\geq k}\}$ to $\mathtt{X}_k$, we follow $\mathcal{P'}^{(k-1)}$ and update the new delay nodes to their ON

states at a suitable step of the path. When $\mathtt{X}_k$ is updated to ON, the new delay nodes are still in their OFF state (because the states where nodes $\{\mathtt{X}_{i\geq k}\}$ are ON are not visited yet), allowing this transition. Then we update the delay node on the self-edge for $\mathtt{X}_k$ to ON. This does not hinder $\mathtt{X}_k$ from turning OFF in order for the system to reach a memory variant of 00..0 in the path. When the path visits a state where any of the variables $\{\mathtt{X}_{j>k}\}$ are ON, update the corresponding delay node to be in the ON state. Since $\mathtt{X}_k$ does not have to be updated again, these delays can be kept until the system reaches $S_{0A}^{(k)}$ (identical to $S_0^{(k-1)}$ but with the new delay nodes in their ON states). Let's call this path $\mathcal{P'}_A^{(k)}$.

Instead, if we add $k-1$ delays on the edges from variables $\{\mathtt{X}_{1<i<k}\}$ to $\mathtt{X}_k$, we follow $\mathcal{P'}^{(k-1)}$, but now keeping the new delay nodes to their OFF states. The new delay nodes do not hinder $\mathtt{X}_k$ from turning ON from 00..0 (because the delay nodes are OFF) nor from turning OFF again (because $\mathtt{!X}_k$ is part of the update function of $\mathtt{X}_k$). Let's call this path $\mathcal{P'}_B^{(k)}$. $\mathcal{P'}_B^{(k)}$ leads to a state $S_{0B}^{(k)}$ that is identical to $S_0^{(k-1)}$ but has the new delay nodes in their OFF states.

Regardless of the choice between $\mathcal{P'}_A^{(k)}$ or $\mathcal{P'}_B^{(k)}$, the system can then follow a path $\mathcal{P}^{(k)}$ almost identical to $\mathcal{P}^{(k-1)}$, leading to $S_{k-1}^{(k)}$ (identical to $S_{k-1}^{(k-1)}$ but with the new delays), as it does not involve the update of $\mathtt{X}_k$. Finally, at state $S_{k-1}^{(k)}$, we turn ON $\mathtt{X}_k$ to transition to $S_k^{(k)}$, either by using the ON states of $N-k+1$ delays coming from $\mathcal{P'}_A^{(k)}$ to use the transition from 11..1, or by using the OFF states of $k-1$ delays coming from $\mathcal{P'}_B^{(k)}$ to use the transition from 00..0.

Claim 4: Always choosing the smaller number of delays for each $\mathtt{X}_k$ gives $\lfloor N^2/4 \rfloor$ delays.

Always choosing the smaller number of delays for any k gives $\sum_{k=2}^{N} min(k-1, N-k+1)$, which is $N^2/4$ for even N and $(N^2-1)/4$ for odd N.

Claim 5: It is not possible to eliminate a star-shaped MAA with fewer delays than those indicated by the algorithm.

Suppose that the star-shaped MAA is eliminated in a system with a certain number of delay nodes. This means that there must exist a path of state transitions from the canonical 00..0 state to the trap space 11..1. For this path to exist, for any $0 \leq k < N$, the path must contain a state S_k (not necessarily unique) wherein k non-delay variables are in their ON states and which has a transition to a state S_{k+1} where $k+1$ non-delay variables are ON. Consider one such transition and suppose, without loss of generality, that node $\mathtt{X}_{k+1}$ turns ON in this transition. In the absence of delays, activating $\mathtt{X}_{k+1}$ requires all inputs to $\mathtt{X}_{k+1}$ to be OFF or for all inputs to $\mathtt{X}_{k+1}$ to be ON. Therefore, in S_k, the variable $\mathtt{X}_{k+1}$ must read either a memory state wherein the delay nodes copying the states of the k non-delay variables that are ON are set to OFF, or a memory state wherein the delay nodes copying the states of the $N-k$ non-delay variables that are OFF are set to ON. In other words, the variable $\mathtt{X}_{k+1}$ must observe either all regulators as ON, or all regulators as OFF to update, but since k non-delay variables are ON, and $N-k$ non-delay variables are OFF, we need to "mask" either of these groups through delays. This requires a minimum of k or $N-k$ delays, respectively, meaning

that any transition from any S_k to any S_{k+1} requires $\min(k, N-k)$ delay nodes on $\mathtt{X}_{k+1}$. Therefore, at least $\sum_{k=0}^{N-1} \min(k, N-k)$ delays must exist for the whole system.

In summary, we have shown via an algorithm that it is possible to eliminate the star-shaped MAA with $\lfloor N^2/4 \rfloor$ delay nodes. We have also shown that it is not possible to destroy the star-shaped MAA using fewer delay nodes. Therefore, the minimum number of delays necessary and sufficient to destroy the star MAA is $\lfloor N^2/4 \rfloor$.

We verified computationally that the formula above correctly indicates the required number of delays by exhaustively trying all delay combinations of size $\lfloor N^2/4 \rfloor - 1$ on star-shaped MAAs for up to $N = 5$.

Illustration of the algorithm and Claims 1-3 for $N = 4$:

In the following we illustrate the elements of the claims above in the case of $N = 4$.

The upper bound algorithm for $N = 4$ indicates that the elimination of the MAA requires 6 delays: $\mathtt{X}_2$ needs delays from $N-1 = 3$ variables $\{\mathtt{X}_2, \mathtt{X}_3, \mathtt{X}_4\}$, $\mathtt{X}_3$ needs delays from $\{\mathtt{X}_3, \mathtt{X}_4\}$, and $\mathtt{X}_4$ needs a delay on its self-regulation. The middle panel of Figure B5 visualizes this delay-expanded system (using an alphabetical variable notation for simplicity). The figure indicates a shortest path between a canonical state derived from the original system's MAA to the trap space. The succession of states in this path is an instance of $\mathcal{P}'_A$ (the first two rows of states) followed by $\mathcal{P}$ (the last row). The first part of $\mathcal{P}'_A$ (the transitions from the canonical 00..0 state to the canonical state with D ON) are not included in this shortest path.

We illustrate the paths $\mathcal{P}$ and $\mathcal{P}'$ in the case that the first delay node to be added, $\mathtt{d}_{1,2}$, is on the edge from from $\mathtt{X}_1$ to $\mathtt{X}_2$, and we are at the step of considering $\mathtt{X}_3$ (i.e., $k = 3$). We indicate the delay-expanded system's states in the order $\mathtt{X}_1$, $\mathtt{d}_{1,2}$, $\mathtt{X}_2$, $\mathtt{X}_3$, $\mathtt{X}_4$. In this case, the path $\mathcal{P}$, which in general connects S_0 to S_{k-1} (the state before updating $\mathtt{X}_k$), is $\mathcal{P}^{(2)}$: (00 0 0 0) $\rightarrow$ (10 0 0 0) $\rightarrow$ (10 1 0 0). The generic $\mathcal{P}'$ path starts at the canonical state 00..0 in the MAA, visits states where each of the nodes is ON individually, going back to a canonical or memory variant of 00..0 each time, and reaches a state S_1 (which has $\mathtt{X}_1 = 1$). In this case, $\mathcal{P}'$ is $\mathcal{P}'^{(2)}_B$: (00 0 0 0) $\rightarrow$ (10 0 0 0) $\rightarrow$ (00 0 0 0) $\rightarrow$ (00 1 0 0) $\rightarrow$ (00 0 0 0) $\rightarrow$ (00 0 1 0) $\rightarrow$ (00 0 0 0) $\rightarrow$ (00 0 0 1) $\rightarrow$ (00 0 0 0).

Next, there are two choices of delay combinations to add to $\mathtt{X}_3$: from $\mathtt{X}_1$ and $\mathtt{X}_2$ (as in Claim 2), or on the self-regulation of $\mathtt{X}_3$ and from $\mathtt{X}_4$ (as in Claim 1). The delay-expanded system for the first choice is indicated in Equation A1, and the delay-expanded system for the second choice is in Equation A2.

```
 X1, (!X1 & !X2 & !X3 & !X4) | (X1 & X2 & X3 & X4)
d1,2, X1
d1,3, X1
 X2, (!d1,2 & !X2 & !X3 & !X4) | (d1,2 & X2 & X3 & X4)
d2,3, X2
 X3, (!d1,3 & !d2,3 & !X3 & !X4) | (d1,3 & d2,3 & X3 & X4)
 X4, (!X1 & !X2 & !X3 & !d4) | (X1 & X2 & X3 & d4)
```

$$\mathtt{d}_4,\ \mathtt{X}_4 \tag{A1}$$

The path $\mathcal{P'}^{(3)}_B$ is a specific instance of the path $\mathcal{P'}$ applied to the case when delay nodes are added from $\mathtt{X}_1$ and $\mathtt{X}_2$, and the state of these delay nodes is OFF. The system state is now given in the order $\mathtt{X}_1$, $\mathtt{d}_{1,2}$, $\mathtt{d}_{1,3}$, $\mathtt{X}_2$, $\mathtt{d}_{2,3}$, $\mathtt{X}_3$, $\mathtt{X}_4$. In this case $\mathcal{P'}^{(3)}_B$ is (000 00 0 0) $\rightarrow$ (100 00 0 0) $\rightarrow$ (000 00 0 0) $\rightarrow$ (000 10 0 0) $\rightarrow$ (000 00 0 0) $\rightarrow$ (000 00 1 0) $\rightarrow$ (000 00 0 0) $\rightarrow$ (000 00 0 1) $\rightarrow$ (000 00 0 0).

$$\begin{aligned}
&\mathtt{X}_1,\ (!\mathtt{X}_1\ \&\ !\mathtt{X}_2\ \&\ !\mathtt{X}_3\ \&\ !\mathtt{X}_4)\ |\ (\mathtt{X}_1\ \&\ \mathtt{X}_2\ \&\ \mathtt{X}_3\ \&\ \mathtt{X}_4)\\
&\mathtt{d}_{1,2},\ \mathtt{X}_1\\
&\mathtt{X}_2,\ (!\mathtt{d}_{1,2}\ \&\ !\mathtt{X}_2\ \&\ !\mathtt{X}_3\ \&\ !\mathtt{X}_4)\ |\ (\mathtt{d}_{1,2}\ \&\ \mathtt{X}_2\ \&\ \mathtt{X}_3\ \&\ \mathtt{X}_4)\\
&\mathtt{X}_3,\ (!\mathtt{X}_1\ \&\ !\mathtt{X}_2\ \&\ \ \&\ \mathtt{d}_3\ \&\ !\mathtt{d}_{4,3})\ |\ (\mathtt{X}_1\ \&\ \mathtt{X}_2\ \&\ \mathtt{d}_3\ \&\ \mathtt{d}_{4,3})\\
&\mathtt{d}_3,\ \mathtt{X}_3\\
&\mathtt{X}_4,\ (!\mathtt{X}_1\ \&\ !\mathtt{X}_2\ \&\ !\mathtt{X}_3\ \&\ !\mathtt{d}_4)\ |\ (\mathtt{X}_1\ \&\ \mathtt{X}_2\ \&\ \mathtt{X}_3\ \&\ \mathtt{d}_4)\\
&\mathtt{d}_{4,3},\ \mathtt{X}_4\\
&\mathtt{d}_4,\ \mathtt{X}_4
\end{aligned} \tag{A2}$$

The path $\mathcal{P'}^{(3)}_A$ is a specific instance of the path $\mathcal{P'}$ applied to the case when delay nodes are added on the self-regulation of $\mathtt{X}_3$ and from $\mathtt{X}_4$, and the state of these delay nodes is ON. The system state is now given in the order $\mathtt{X}_1$, $\mathtt{d}_{1,2}$, $\mathtt{X}_2$, $\mathtt{X}_3$, $\mathtt{d}_3$, $\mathtt{X}_4$, $\mathtt{d}_{4,3}$. In this case $\mathcal{P'}^{(3)}_A$ is (00 0 00 00) $\rightarrow$ (10 0 00 00) $\rightarrow$ (00 0 00 00) $\rightarrow$ (00 1 00 00) $\rightarrow$ (00 0 00 00) $\rightarrow$ (00 0 10 00) $\rightarrow$ (00 0 11 00) $\rightarrow$ (00 0 01 00) $\rightarrow$ (00 0 01 10) $\rightarrow$ (00 0 01 11) $\rightarrow$ (00 0 01 01). This path involves the canonical 00..0 state until the delay node on the self-regulation of $\mathtt{X}_3$ is updated and involves memory states afterwards.

The bottom panel of Figure B5 indicates an example of a shortest path from a canonical state derived from the original system's MAA to the trap space 11..1 in the case of the system of Equation A1. This path starts with the minimal necessary step of $\mathcal{P'}^{(4)}_B$, namely the start from the canonical state with $D = 1$, then turning D OFF, it is followed by $\mathcal{P}^{(4)}$, namely the succession of memory states that turn A, then B, then C, then D ON, and ends with the delay nodes turning ON as well.

In a confirmation of the algorithm and the claims, we found that the $N = 4$ systems with the minimal combinations of delays that no longer have an MAA are $N! = 24$ permutations of Equation A1 and 24 permutations of Equation A2. These combinations of delays are the two possibilities of minimal delay combinations indicated by the algorithm. For $N = 5$ we verified that no combinations of five delays can eliminate the MAA. We also verified that the 6-delay combination indicated by the algorithm, with delays on the edges from $\mathtt{X}_1$ to $\mathtt{X}_2$, from $\mathtt{X}_1$ to $\mathtt{X}_3$, from $\mathtt{X}_2$ to $\mathtt{X}_3$, the self-edge on $\mathtt{X}_4$, the edge from $\mathtt{X}_5$ to $\mathtt{X}_4$, and the self-edge of $\mathtt{X}_5$, successfully eliminates the MAA.

A.3 Not all memory states are reachable from canonical states

As we described in the main text, the canonical states of a delay-expanded system retain the reachability of the original system. Yet, a non-canonical (memory) state may gain a transition compared to the original system (i.e., the delay-affected variable changes state although its state stays the same in the original system) or lose a transition (i.e., the delay-affected variable stays the same even though it changes state in the original system).

Lost transitions of memory states can interfere with the capacity of gained transitions to eliminate the MAA. For example, even if a memory state gains a transition that connects it to the trap space, this memory state may not be reachable from the canonical states of the motif-avoidant attractor because another memory state has lost a transition. In these cases of unreachability the motif-avoidant attractor is preserved. We identified that this unreachability is not avoidable in general, except for memory states arising from the linear extension of a self-edge. In this case, the canonical states have transitions to memory states whenever the delay-affected variable (which is also the delay node's parent node) is updated. Since the connectivity of canonical states is preserved, these memory states take part in the strongly connected component of the state transition graph as the mediators of paths between canonical states. When the variables unaffected by delays are updated, all memory states retain the original connectivity. This ensures that all memory states of the MAA are reachable when self-edges are extended.

An example of a failed MAA elimination due to an unreachable memory state is offered by the system in Example 4, and is illustrated in Figure A1. The MAA visits all three states that are at Hamming distance 1 from the trap space 111, thus turning a single variable ON from a system state in which the other two variables are already ON should be a viable strategy to eliminate the MAA. A possible way to turn variable `A` ON in a memory version of the 011 state is to add a delay on the `C` to `A` edge. This edge is not part of the regulations that maintain the trap space. Such a delay allows `A` to turn ON from the memory state 0110 (see the light blue edge) and creates a path to the trap space. However, the memory state 0110 is not accessible from the strongly connected component of the state transition graph because the memory version of the state 101 (1010) lost its transition to the memory version of 001 (0010), which existed in the original system (compare the light blue edge among memory states to the bold black edge among canonical states). As a consequence, the motif-avoidant attractor is preserved. Another way to turn variable `A` ON in a memory version of the 011 state is to add a delay to the self-regulation of `A`, which is part of the stable motif, and use the memory of a past ON state of `A`. The projected state transition graph for that delay is indicated in Figure 5. Here we indicate the equivalent scenario of adding a delay to the self-edge of `C`, which also is part of the regulations that maintain the trap space. The delay leads to two new state transitions in the delayed system, including a transition from the memory state 1101 to the trap space 1111. The memory state 1101 is part of the strongly connected component of states in the delayed system, thus the new transition places the trap space in the out-component of the strongly connected component. As the out-component is not empty, there no longer is a motif-avoidant attractor. In summary, the unreachability issue makes the delay on the `C` to `A` edge

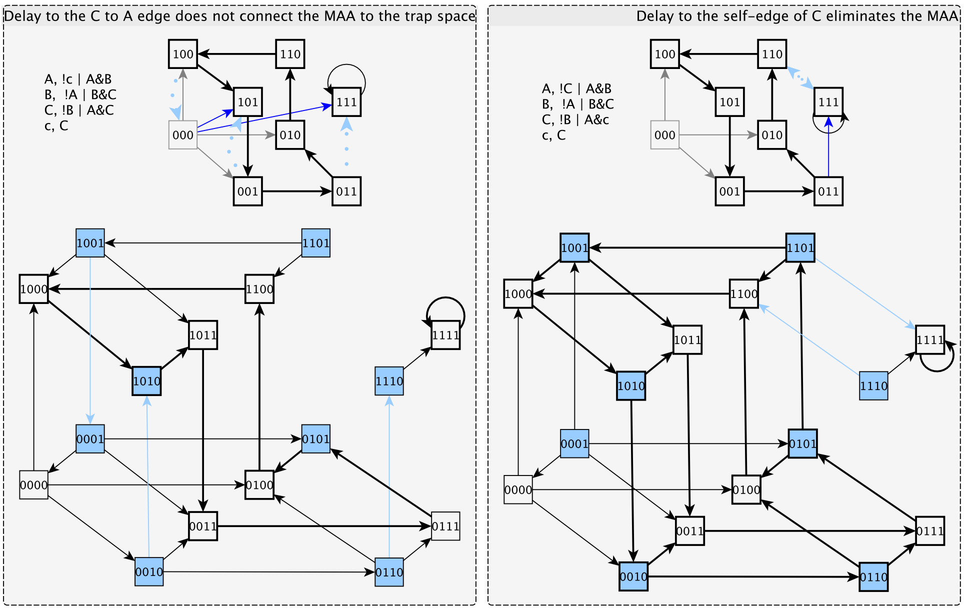

Fig. A1: Illustration of the unreachability of certain memory states from canonical states. We expand the system in Example 4 with a delay to the inhibition of `A` by `C` (left panel) or a delay to the self-regulation of `C`. For both cases, we indicate the full state transition graph as well as the projected state transition graph. In the state transition graph of the delayed systems, the canonical states have white background and the memory states have light blue background. The state transitions that are different for memory states compared to canonical states are marked in light blue in both the full and projected state transition graphs. In each panel the strongly connected components of states corresponding to the original (delay-free) system's motif-avoidant attractor is indicated with thick edges and thick node outlines. Each dark blue edge of the projected state transition graph arises from a path among canonical states in the delayed system.

unable to eliminate the MAA, but a delay on the self-regulation of any of the three variables is able to eliminate the MAA.

Notably, the example illustrates that memory states lose transitions only when the delay-affected variable is updated. Our algorithm for the general upper bound and the specific solution for the star-shaped MAA avoid the unreachability issue by identifying a path that allows all necessary delay nodes to be updated to the needed states *after* the delay-affected variable is updated to its state that is opposite of the trap space. The delay nodes can retain these states until the delay-affected variable is updated again, and allow it to align with the trap space.

At present there is no estimate of the likelihood of a memory state needed to create a path to the trap space being unreachable from the motif-avoidant attractor. We observed that unreachable memory states are fairly common, but this unreachability

K\N	4	5	6	7	8	9	10
2	3.8e5	6.3e5	9.3e5	12.5e5	17.5e5	21.0e5	27.3e5
3	2.2e5	3.5e5	5.1e5	7.2e5	9.8e5	11.4e5	14.8e5
4	1.0e5	2.0e5	3.0e5	3.8e5	5.2e5	6.5e5	8.1e5
5	-	1.0e5	1.4e5	2.0e5	2.4e5	3.3e5	4.2e5
6	-	-	0.8e5	1.1e5	1.4e5	1.7e5	2.1e5
7	-	-	-	0.7e5	0.9e5	1.1e5	1.3e5
8	-	-	-	-	0.6e5	0.7e5	1.0e5
9	-	-	-	-	-	0.6e5	0.7e5
10	-	-	-	-	-	-	0.6e5
21 301 660 total networks							

(a) Small networks

K\N	20	30	40
2	11.1e6	25.4e6	78.1e6
3	4.9e6	9.9e6	16.3e6
4	2.6e6	4.7e6	1.9e6
5	1.2e6	2.4e6	0.3e6
158 948 231 total networks			

(b) Large networks

Table B1: The number of sampled random networks across N-K ensembles. For the small networks, each ensemble was sampled until 1000 networks with motif-avoidant attractors were detected. The same criterion was implemented for the large networks, but the [N=40, K=4] and [N=40, K=5] ensembles had to be terminated prematurely, because not enough motif-avoidant attractors were discovered, even after several weeks of runtime.

rarely prevents a path to the trap space. We have tried and failed to find examples of unsuccessful MAA elimination due to unreachability in cases where $m > 1$. (Recall that $m < d$ represents the minimal disagreement between a state of the MAA with the fixed variables of the trap space.) In all of our examples, we observed that multiple combinations of delays could create paths to the trap space, and only a subset of these paths were affected by unreachability issues. At least one path remained that did successfully eliminate the MAA.

If in fact it is true that unreachability only occurs for $m = 1$, then this issue does not pose a problem to MAA elimination. In the case of $m = 1$, the MAA can be eliminated by adding a delay node to a self-regulation. For different values of m, delays on edges within the MAA could be used to lower the number of delays needed to eliminate the MAA.

Appendix B Supplementary Figures

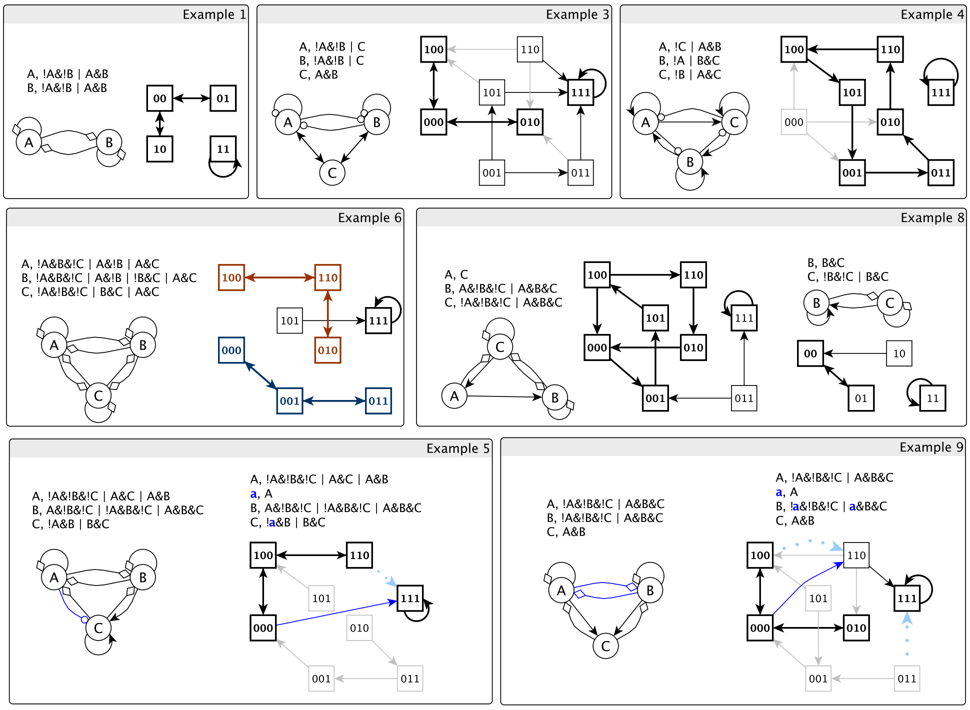

Fig. B2: The interaction graphs and state transition graphs of the systems in Examples 1, 3-6, 8 and 9. In the interaction graph a terminal arrow indicates a positive edge, a terminal circle indicates a negative edge and a terminal diamond indicates a dual edge. In the state transition graph the attractors are indicated in bold outlines. The interaction graphs of Examples 5 and 9 indicate in blue the edges whose linear extension eliminates the motif-avoidant attractor. We indicate the projected state transition graph corresponding to one linear extension. Example 5 illustrates that escaping the subspace of the MAA can be accomplished by linear extension of a subset of the edges incident on the variables fixed by the MAA. Example 9 illustrates that when the state that escapes the subspace can reach the trap space, it is enough to use delays that allow reaching this escape state, and no delays to the fixed node(s) are needed.

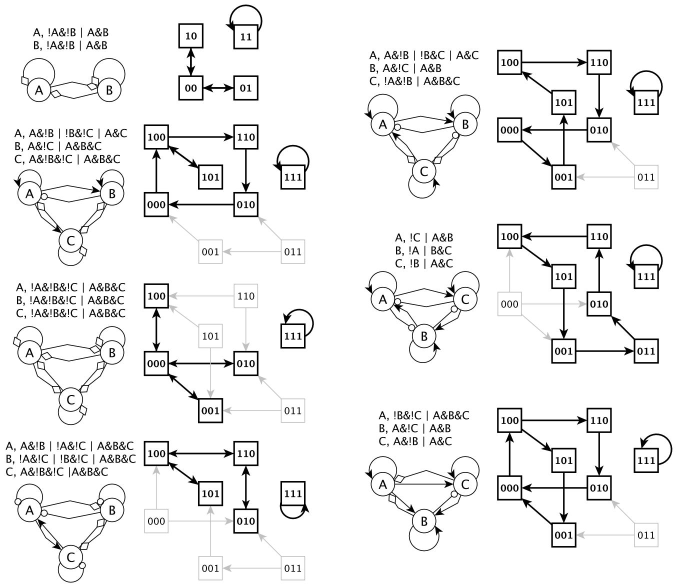

Fig. B3: Prototypical (template) MAAs with minimal connectivity in the state transition graph (i.e. eliminating any state transition would eliminate the MAA). For each MAA we indicate the interaction graph, the set of Boolean functions, and the STG of a system that also has a point attractor 111.

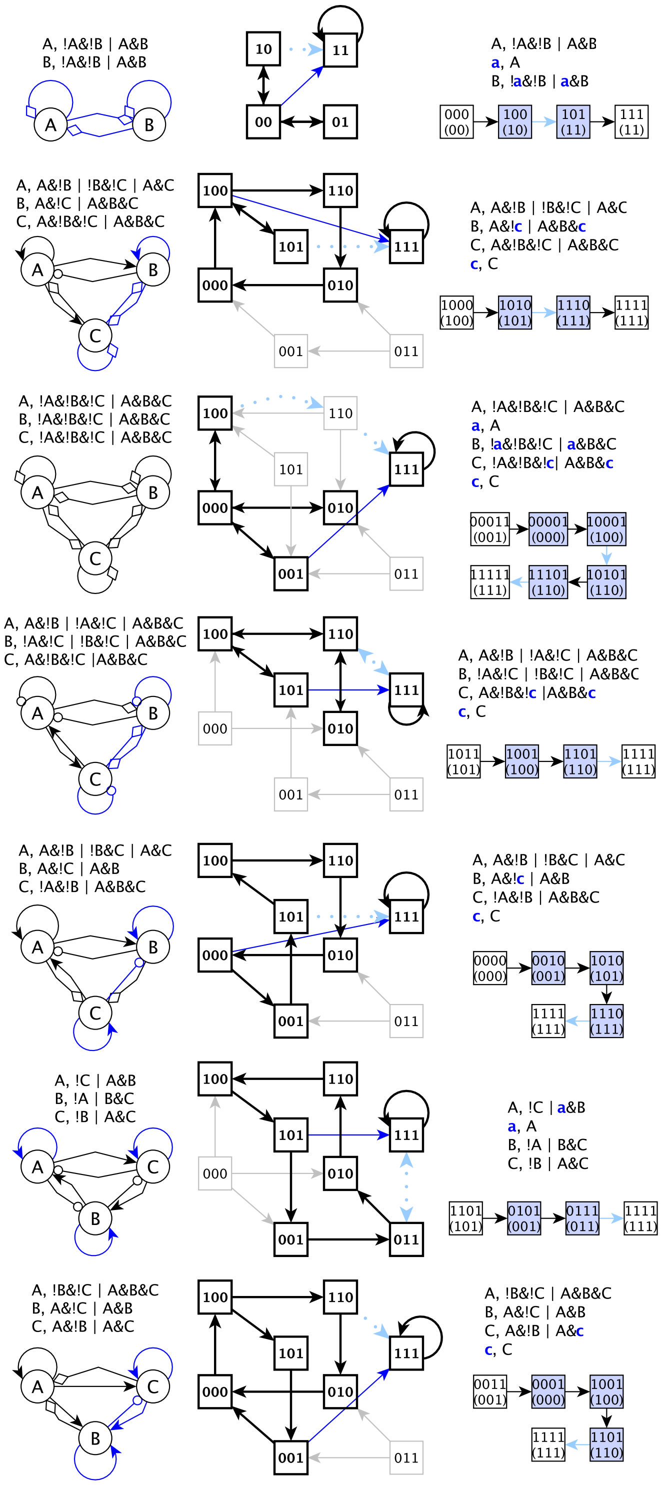

Fig. B4: The prototypical (template) MAAs are easy to eliminate. For each MAA we indicate the interaction graph, the set of Boolean functions, and the projected STG of a system that also has a point attractor 111. All but one MAA have single delays that eliminate the MAA; the edges whose linear extension eliminates the MAA are shown in blue in the interaction graph. For each MAA we indicate one example of a single (or in one case, double) delay that eliminates the MAA. In the projected STGs the states and state transitions that make up attractors are shown in bold. The state transitions due to the delay nodes (and involving memory states) are shown with dotted light blue edges. The blue edges among canonical states arise from paths of state transition in the delayed system; these paths are indicated below the projected STG and highlight the state of each delay node in blue.

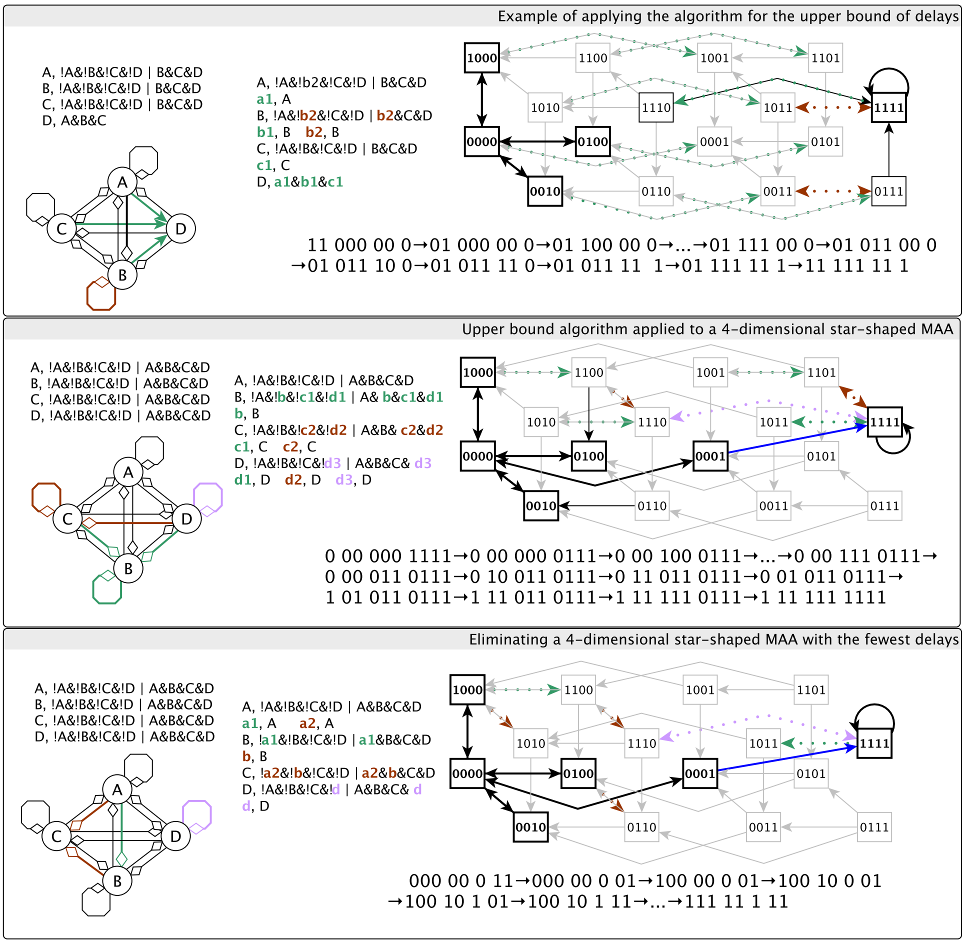

Fig. B5: Illustration of the motif-avoidant attractor elimination algorithm and the algorithm that applies specifically to star-shaped MAAs as in Example 10. The visualization of interaction graphs and projected STGs is the same as in previous figures. The edges of the interaction graphs are colored by the sequence of variables to turn ON. Top panel: A four-variable system with $d = n = 3$, $m = 1$. As D has a fixed state in the MAA, the first step is to add delays to the edges from A, B, and C to D (green); these allow escape from the $**{*}0$ subspace. A delay on the self-regulation of B (purple) allows B to turn ON in a memory version of 0011, creating a path to the trap space. The middle and bottom panels compare the algorithm that uses the transitions of the trap space with one possible application of the specific algorithm presented in Appendix A on a 4-variable star-shaped MAA. An alternative possibility for the latter is to replace the delays on the edges from A to C and from B to C with delays on the edge from D to C and the self-regulation of C (see Eq. A2).
